# PRMT1 Ablation in Endothelial Cells Causes Endothelial Dysfunction and Aggravates COPD Attributable to Dysregulated NF‐κB Signaling

**DOI:** 10.1002/advs.202411514

**Published:** 2025-03-26

**Authors:** Thi Thuy Vy Tran, Yideul Jeong, Suwoo Kim, Ji Eun Yeom, Jinwoo Lee, Wonhwa Lee, Gyu‐Un Bae, Jong‐Sun Kang

**Affiliations:** ^1^ Department of Molecular Cell Biology Sungkyunkwan University School of Medicine 2066, Seobu‐Ro, Jangan‐gu Suwon Gyeonggi‐do 16419 Republic of Korea; ^2^ Research Institute of Aging Related Disease AniMusCure Inc. Suwon 16419 Republic of Korea; ^3^ Department of Chemistry Sungkyunkwan University Suwon 16419 Republic of Korea; ^4^ Drug Information Research Institute Muscle Physiome Research Center College of Pharmacy Sookmyung Women's University Cheongpa‐ro 47‐gil 100, Yongsan‐gu Seoul 04310 Republic of Korea

**Keywords:** COPD, endothelial dysfunction, NF‐κB, PRMT1, senescence

## Abstract

Endothelial dysfunction and senescence are pivotal in pulmonary diseases, including chronic obstructive pulmonary disease (COPD). Protein arginine methyltransferase 1 (PRMT1) is the major enzyme responsible for asymmetric arginine dimethylation and plays a role in diverse biological processes, including cardiovascular function. Yet, its role in endothelial cells (ECs) remains poorly understood. Here, the role of PRMT1 is investigated in ECs, particularly in the context of COPD pathogenesis. Endothelial‐specific PRMT1 knockout mice exhibit pulmonary hemorrhage, inflammation, barrier disruption, and apoptosis, accompanied by hyperactivation of nuclear factor kappa B (NF‐κB). Bulk RNA sequencing of whole lungs and single‐cell RNA sequencing of pulmonary ECs reveal that endothelial PRMT1 ablation results in a major alteration in inflammation‐related gene expression. In a COPD model, PRMT1 deficiency aggravates the COPD phenotypes, including enlarged alveolar spaces, increased cell death, and senescence. PRMT1 inhibition in ECs exacerbates tumor necrosis factor alpha‐triggered EC senescence and dysfunction attributable to NF‐κB hyperactivation. PRMT1 as a critical regulator of pulmonary EC function, preventing NF‐κB‐driven endothelial dysfunction and senescence is highlighted here.

## Graphical abstract

This study investigates the role of protein arginine methyltransferase 1 (PRMT1) in endothelial cells (ECs) in chronic obstructive pulmonary disease (COPD). Mice with endothelial‐specific PRMT1 deletion develop pulmonary hemorrhage, inflammation, and apoptosis, driven by excessive nuclear factor kappa B activation. PRMT1 deficiency aggravates COPD by promoting EC senescence and dysfunction, highlighting its role in maintaining endothelial homeostasis and vascular stability.

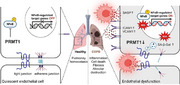

## 1 Introduction

Chronic obstructive pulmonary disease (COPD) is a prevalent and progressive lung disease characterized by chronic lung inflammation and irreversible alveolar destruction. COPD is known as a leading cause of death worldwide, with limited therapeutic options to prevent disease progression.^[^ ^1^ ^]^ Endothelial dysfunction, characterized by impaired endothelial barrier function and a proinflammatory phenotype, is increasingly recognized as a hallmark contributor to COPD pathogenesis and progression.^[^ ^2^, ^3^ ^]^ Endothelial senescence, characterized by growth arrest coupled with a proinflammatory secretion, is known as senescence‐associated secretory phenotype and activation of β‐Galactosidase.^[^ ^4^, ^5^ ^]^ Despite the established role of endothelial dysfunction in COPD, the molecular mechanisms that maintain endothelial cell (EC) function and the pathways through which dysregulation leads to endothelial senescence in the disease remain poorly understood.

The shift toward proinflammatory characteristics associated with EC dysfunction hinges on the activation of nuclear factor kappa B (NF‐κB), which serves as a central switch in this pathological process.^[^ ^6^ ^]^ In quiescent cells, NF‐κB exists in an inactive form within the cytoplasm, forming complexes with inhibitor of κB (IκB) proteins.^[^ ^7^ ^]^ Upon exposure to external stimuli, including tumor necrosis factor alpha (TNF‐α), interleukin‐1 (IL‐1), reactive oxygen species, Angiotensin II, or other activators, NF‐κB is released from the IκB complex. This release is driven by the degradation of IκB, leading to the nuclear translocation of NF‐κB where it activates target genes.^[^ ^6^ ^]^ Identifying these triggers and understanding the molecular mechanisms governing NF‐κB activation are crucial for developing strategies to restrain NF‐κB activity and prevent the onset of endothelial dysfunction.

Protein arginine methyltransferases (PRMTs) are enzymes that catalyze the transfer of a dimethyl group to arginine residues of target proteins. Protein arginine methyltransferase 1 (PRMT1) is a major cellular PRMT that controls a wide range of tissue homeostasis including proliferation/survival and death of stem cells, DNA damage response, hepatic gluconeogenesis, and cardiovascular function.^[^ ^8^ ^]^ Studies have shown that the loss of PRMT1 specifically in cardiomyocytes leads to dilated cardiomyopathy and heart failure.^[^ ^9^ ^]^ In addition, PRMT1 inhibition by gene ablation or inhibitor treatment in cardiomyocytes results in endoplasmic reticulum stress response leading to increased sensitivity to doxorubicin and cardiomyocytes death.^[^ ^10^, ^11^ ^]^ Furthermore, PRMT1 has a protective function in vascular smooth muscle, as deleting PRMT1 in these cells leads to aortic dissection, elastic fiber degeneration, and cell death.^[^ ^12^ ^]^ PRMT1 has also been associated with vascular diseases through heightened production of asymmetric dimethylarginine, an endogenous inhibitor of nitric oxide synthase.^[^ ^13^ ^]^ A recent study suggested a potential role of PRMT1 in the control of p65/NF‐κB activity through arginine dimethylation.^[^ ^14^ ^]^ However, the role of PRMT1 in ECs is currently unclear. The initial expression analysis for PRMT1 suggested that PRMT1 specifically declined in ECs among pulmonary cell types of COPD samples. Thus, we examined the significance of PRMT1 in EC homeostasis and its involvement in the development and progression of COPD.

Hereto, we generated mice with tamoxifen‐inducible PRMT1 deletion specifically in ECs, controlled by the *Cdh5*/ERT2‐Cre recombinase. Strikingly, the endothelial PRMT1 knockout (KO) mice developed pulmonary hemorrhage associated with EC dysfunction, characterized by increased inflammation and NF‐κB activation. Consistently, a single copy deletion of PRMT1 in ECs exacerbated COPD phenotypes related to enhanced inflammation and NF‐κB activation. Thus, PRMT1 is essential for maintaining pulmonary ECs homeostasis, through suppression of NF‐κB signaling.

## 2 Results

### 2.1 PRMT1 Expression Is Distinctively Declined in Pulmonary ECs from COPD Patients and Mouse Models

The expression of *PRMT1* across various tissues and cell types using RNA sequencing data from the Human Protein Atlas‐derived GTEx datasets, revealing widespread expression, with notably high levels detected in the testis and lung tissue (Figure S1A, Supporting Information). Furthermore, ECs exhibited the highest levels of *PRMT1* expression among pulmonary cell populations in human lung tissues. To further investigate the expression of *PRMT1* and other *PRMT* family members in pulmonary cell types, we analyzed Gene Expression Omnibus (GEO) database from both human (GSE173896) and mouse (GSE168299) lung.^[^ ^15^, ^16^ ^]^ This analysis revealed that *PRMT1* expression is most prominent in general capillary ECs (gCap) (Figure S1B,C, Supporting Information).

**Figure 1:**
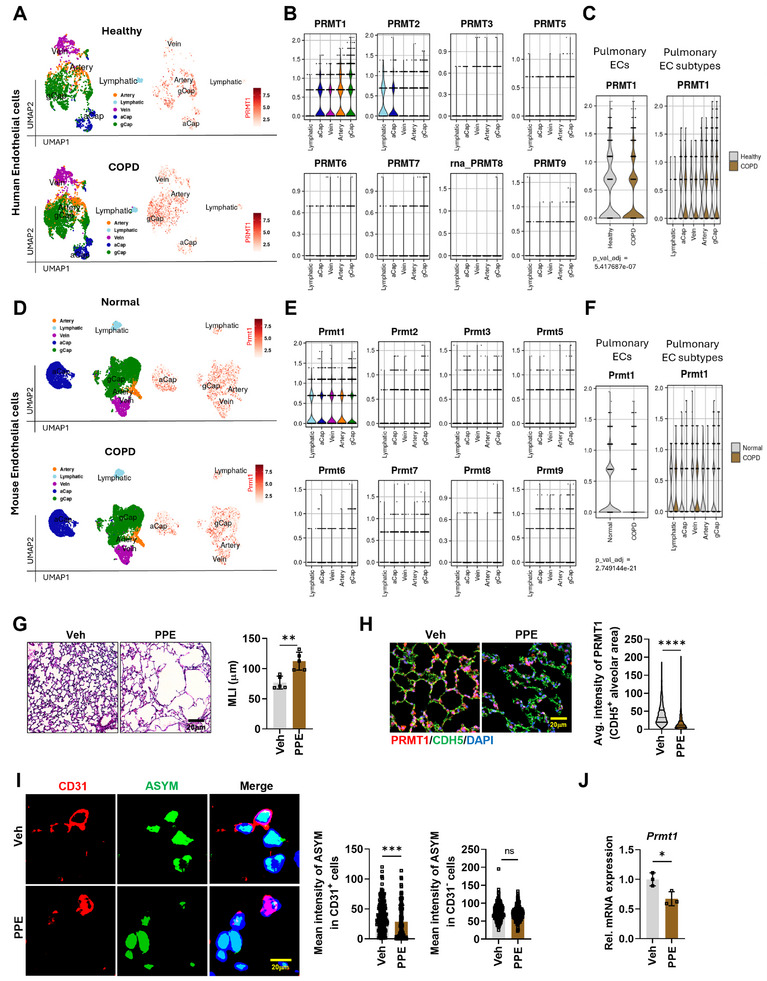
PRMT1 is distinctively declined in pulmonary ECs from COPD patients and COPD mouse model. A) Uniform Manifold Approximation and Projection (UMAP) representation of pulmonary ECs from healthy or COPD human samples derived from GEO database (GSE173896). B) Violin blot analysis for gene expression of *PRMT1* or other *PRMT*s in five EC subpopulations. C) Violin blot analysis for comparing expression of *PRMT1* in total pulmonary ECs (left) and in five EC subpopulations (right) in healthy and COPD cohorts. D) UMAP presentation of ECs from the lung of normal or COPD mouse samples derived from GEO database (GSE168299). E) Violin blot analysis for gene expression of *Prmt1* or other *Prmt*s in five EC subpopulations. F) Violin blot analysis for comparing expression of *Prmt1* in total pulmonary ECs (left) and in five EC subpopulations (right) in normal and COPD mice. G) Histological analysis of lungs from vehicle (Veh) or PPE‐treated (PPE) mice. Scale bar = 20 µm. Quantification of mean linear intercept (MLI) of lungs. H) Confocal images of alveolar area, stained for PRMT1 (red), CDH5 (green), and counterstained with 4′,6‐diamidino‐2‐phenylindole (DAPI) (blue). Scale bar = 20 µm. Quantification of PRMT1 expression intensity in CDH5^+^ alveolar areas. I) Confocal images of pulmonary cells stained for ECs (CD31, red), ASYM (green), and counterstained with DAPI (blue). Scale bar = 20 µm. Quantification of mean intensity of ASYM in CD31‐positive cells and CD31‐negative cells. J) Quantitative reverse transcription polymerase chain reaction (qRT‐PCR) analysis of *Prmt1* using total lung RNA from Veh or PPE mice (n = 3). Data for all panels are means ± standard deviation (SD). Student's *t*‐test. ns = not significant, *p < 0.05, **p < 0.01, ***p < 0.001, ****p < 0.0001.

To investigate the involvement of PRMT1 in COPD, we analyzed the expression levels of *PRMTs*, using GEO database from normal and COPD patients (GSE57148).^[^ ^17^ ^]^ We found that *PRMT1* and *PECAM1*, a well‐known marker for ECs, are reduced in lungs from COPD patients, while other *PRMT*s showed no detectable expression or significant differences (Figure S1D, Supporting Information). Using single‐cell transcriptomic analysis of published GEO datasets from both healthy individuals and COPD patients (GSE173896),^[^ ^15^ ^]^ as well as control and COPD mouse models (GSE168299),^[^ ^16^ ^]^ we identified five EC populations based on previously established markers:^[^ ^18^ ^]^ artery, lymphatic, vein, aerocyte (aCap), and gCap (Figure S2, Supporting Information, and **Figure** **1**A–F). Among the *PRMT* family members, *PRMT1* showed the highest expression across EC populations, particularly within gCap. Notably, a reduction in *PRMT1* levels was observed in total pulmonary ECs, as well as in specific EC subtypes, including gCap, in the COPD cohorts. Given that COPD is associated with accelerated lung tissue aging,^[^ ^19^ ^]^ we further examined *Prmt1* expression in the context of aging by analyzing previously published single‐cell sequencing (scRNAseq) datasets from young and aged mouse lungs (Figure S3A–E, Supporting Information).^[^ ^20^ ^]^ This analysis revealed a reduction of *Prmt1* expression in aged lungs, with the most decrease observed in ECs, whereas other *Prmt*s generally showed low expressions with no significant changes between young and aged mice.

To confirm the expression of PRMT1 in COPD, we generated a COPD model by injecting a single dose of porcine pancreatic elastase (PPE) to 8‐week‐old mice and analyzed the pulmonary phenotype post 21 d of injection (Figure S4A, Supporting Information). PPE‐injected mice exhibited lung damage, including a notable enlargement of alveolar space, and higher macrophage infiltration, compared to vehicle‐treated mice (Figure S4B,C, Supporting Information, and Figure 1G). Notably, in the PPE‐treated mice, ECs in the alveolar area showed a significant decline in PRMT1 protein and the asymmetrically dimethylated arginine (ASYM) levels (Figure 1H,I). We also confirmed the reduction of *Prmt1* level using total lung mRNA samples (Figure 1J). These findings imply a potential role of PRMT1 in ECs during COPD pathogenesis.

### 2.2 Induced PRMT1 Ablation in ECs Results in Pulmonary Hemorrhage, Associated with Disrupted Cellular Junction, Increased Cell Death, and Inflammation

**Figure 2:**
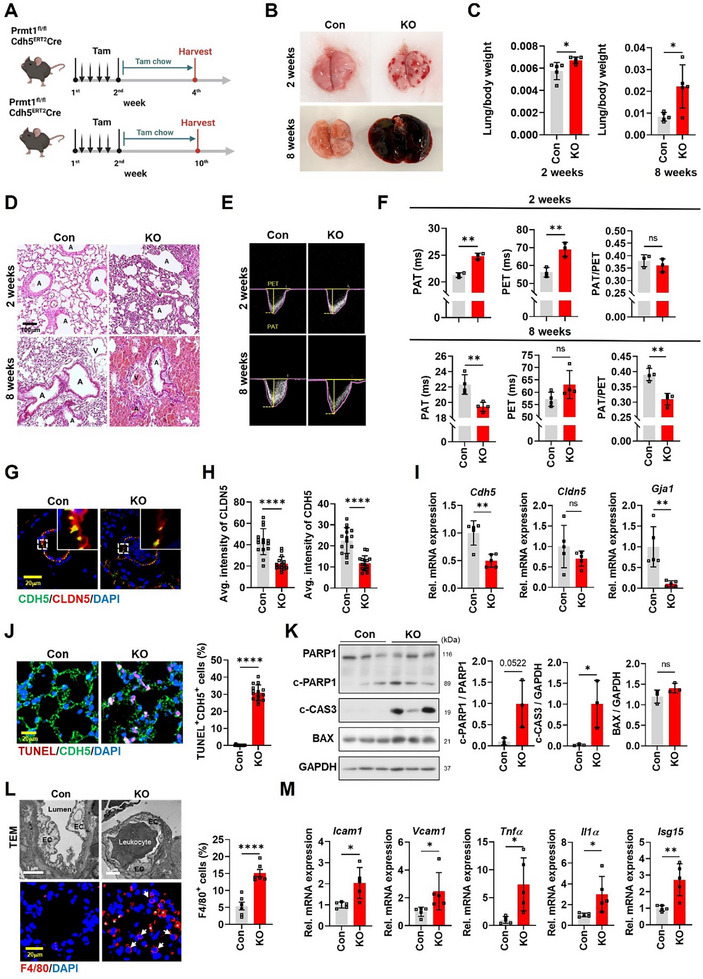
Induced PRMT1 ablation in ECs results in aggravated pulmonary hemorrhage associated with disrupted endothelial junction, increased cell death and inflammation. A) Scheme of tamoxifen injection. 8 weeks‐old *Prmt1* ^fl/fl^ *Cdh5* ^ERT2–Cre^ mice were intraperitoneal administrated with vehicle or tamoxifen (Tam) for 1 week and were fed with tamoxifen‐containing chow (Tam chow) until harvest day. Black arrows, days with injection. B) Photographs of isolated lungs from Con or 2‐week and 8‐week KO mice. C) Evaluation of the relative lung mass to body weight ratio in Con or 2‐week and 8‐week KO mice (*n* = 5). D) Histological analysis of lung tissues using hematoxylin and eosin (H&E) staining in Con or 2‐week and 8‐week KO mice. Scale bar = 100 µm. E) Snapshot of pulsed wave (PW) Doppler showing pulmonary artery flow in Con or 2‐week and 8‐week KO mice. F) Electrocardiographic assessment of pulmonary acceleration time (PAT), pulmonary ejection time (PET), and ratio of PAT to PET in Con or 2‐week (*n* = 3) and 8‐week KO mice (*n* = 4). G) Confocal images of pulmonary vessel in Con or 2‐week KO mice, stained for adherens junction (CDH5, green), tight junction (CLDN5, red), and counterstained with DAPI (blue). Scale bar = 20 µm. H) Quantification of average intensity of CLDN5 and CDN5 in pulmonary vessel of Con or 2‐week KO mice. I) qRT‐PCR analysis of junctional markers, including *Cdh5*, *Cldn5*, and *Gja1* in the lungs of Con or 2‐week KO mice (*n* = 5). J) Confocal images of lung TUNEL (red) costaining with CDH5 (green), and counterstained with DAPI (blue) in Con or 2‐week KO mice. Scale bar = 20 µm. Quantification of the percentage of TUNEL‐positive CDH5‐positive cells. K) Immunoblot analysis for protein expression levels of apoptosis markers, including PARP1, cleaved PARP1 (c‐PARP1), cleaved Caspase 3 (c‐CAS3), BAX, and loading control glyceraldehyde‐3‐phosphate dehydrogenase (GAPDH) of Con and 2‐week KO mice (*n* = 3). Quantification of relative protein level of c‐PARP1 to PARP1, c‐CAS3, and BAX to GAPDH. L) TEM photographs of pulmonary microvessels in Con and 2‐week KO mice (upper panel) and confocal images of lung tissues stained for macrophage marker (F4/80, red) and DAPI (blue) (lower panel). Scale bar = 20 µm. White arrow: F4/80^+^ macrophage, white asterisk: red blood cell. Quantification of percentage of F4/80^+^ cells in Con and 2‐week KO lungs. M) qRT‐PCR analysis of *Icam‐1*, *Vcam‐1*, *Tnf‐α*, *Il‐1α*, and *Isg‐15* using total lung RNA from Con and 2‐week KO mice (*n* = 5). Data for all panels are means ± SD. Student's *t*‐test. ns = not significant, *p < 0.05, **p < 0.01, ***p < 0.001, ****p < 0.0001.

To examine the role of PRMT1 in ECs, we generated mice with inducible EC‐specific ablation of PRMT1 mediated by *Cdh5* ^ERT2–Cre^ recombinase (*Prmt1* ^fl/fl^; *Cdh5* ^ERT2–Cre^). To induce the PRMT1 ablation, 8‐week‐old mice were injected with tamoxifen for a week and subsequently fed with tamoxifen‐containing chow (**Figure** **2**A). The immunostaining, qRT‐PCR, and immunoblotting for PRMT1 expression collectively confirmed the depletion of PRMT1 in CDH5‐positive ECs and a reduction in *Prmt1* levels in KO lungs (Figures S5A–C, Supporting Information). PRMT1 ablation in ECs (KO) for 2 weeks displayed a mild phenotype of pulmonary hemorrhage and lung mass increase compared to vehicle‐treated *Prmt1* ^fl/fl^; *Cdh5* ^ERT2–Cre^ (Con) mice (Figure 2B,C). These phenotypes were greatly exacerbated in 8‐week KO mice. Histological analysis of lung tissues unveiled a progressively worsening blood leakage of KO lungs (Figure 2D).

Given that lung damage can affect cardiac function, including left ventricle (LV) function,^[^ ^21^, ^22^ ^]^ we conducted echocardiographic assessment of cardiac function. Parameters such as ejection fraction (EF) and fraction shortening (FS), exhibited no differences in 2‐week KO mice but showed a declining trend in 8‐week KO mice, although these changes did not reach statistical significance (Figure S5D, Supporting Information). Interestingly, 8‐week KO mice also exhibited significant alterations in pulmonary acceleration time (PAT) and the PAT to pulmonary ejection time (PET) ratio, while 2‐week KO mice showed no significant changes compared to Con mice (Figure 2E,F). These findings collectively suggest an essential role for PRMT1 in the maintenance of pulmonary ECs and vascular functions.

Due to the manifestation of a severe pulmonary phenotype in 8 week‐KO mice, we used 2 week‐KO mice to study the phenotypes and underlying mechanisms of endothelial dysfunction. Immunostaining for adherens junction marker CDH5 and tight junction marker CLDN5 revealed altered endothelial junctions characterized by irregular and weakened staining patterns (Figure 2G,H). Despite these changes, no differences were observed in vessel size (Figure S6A, Supporting Information). Furthermore, both mRNA and protein levels of key junctional markers, including CDH5, CLDN5, and GJA1, were reduced in the KO lungs, compared to Con lungs (Figure 2I and Figure S6B, Supporting Information). Consistent with these findings, transmission electron microscopy (TEM) demonstrated a disrupted junction in KO ECs, unlike a tightly formed cell junction in Con EC (Figure S6C, Supporting Information). Similarly, PRMT1 depletion in HUVECs (shPRMT1) using a shRNA‐adenoviral system for 48 h led to disrupted junctions, in contrast to the tightly formed junctions observed in control‐infected HUVECs (Con) (Figure S6D, Supporting Information). This morphological disruption in PRMT1‐depleted HUVECs was accompanied by a reduction in both mRNA and protein levels of junctional markers, including CLDN5, CDH5, and GJA1 (Figure S6E,F, Supporting Information).

Perturbation of endothelial junctions is often associated with cell death, leading to increased vascular permeability, inflammatory responses, and compromised vascular integrity.^[^ ^23^, ^24^ ^]^ Consistent with this, KO lungs exhibited an accumulation of terminal deoxynucleotidyl transferase dUTP nick end labeling (TUNEL)‐positive apoptotic cells (Figure 2J). Furthermore, both KO lungs and PRMT1‐depleted HUVECs (shPRMT1) exhibited elevated levels of apoptotic‐related proteins, including cleaved PARP1 (c‐PARP1), cleaved Caspase 3 (c‐CAS3), and Bcl‐2‐associated X protein (BAX), compared to their respective control groups (Figure 2K and Figure S6G, Supporting Information). Furthermore, *Bax* transcription was upregulated, compared to Con lungs (Figure S6H, Supporting Information). These findings collectively propose that PRMT1 ablation in ECs elicits a perturbation in EC junctional organization and cell death, thereby contributing to endothelial dysfunction.

TEM analysis showed leukocyte recruitment in vascular lumen of KO lungs while Con lungs a clear vascular lumen was observed in Con lung (Figure 2L). Consistently, KO lungs showed a marked accumulation of macrophages, compared to the Con lungs. Additionally, the mRNA expression levels of inflammatory markers, including *Icam‐1*, *Vcam‐1*, *Tnf‐α*, *Il1‐α*, and *Isg‐15*, were notably elevated in the KO lungs (Figure 2M). Furthermore, KO lungs exhibited an increased expression of *Col1a1* and *Col3a1* compared to Con lungs (Figure S6I, Supporting Information). These data indicate that the ablation of endothelial PRMT1 results in EC dysfunction associated with fibrosis and inflammation.

### 2.3 Transcriptome Analysis Reveals That Endothelial PRMT1 Ablation Causes Up‐ and Downregulation of Genes Related to Immune Response and Developmental Morphogenesis

**Figure 3:**
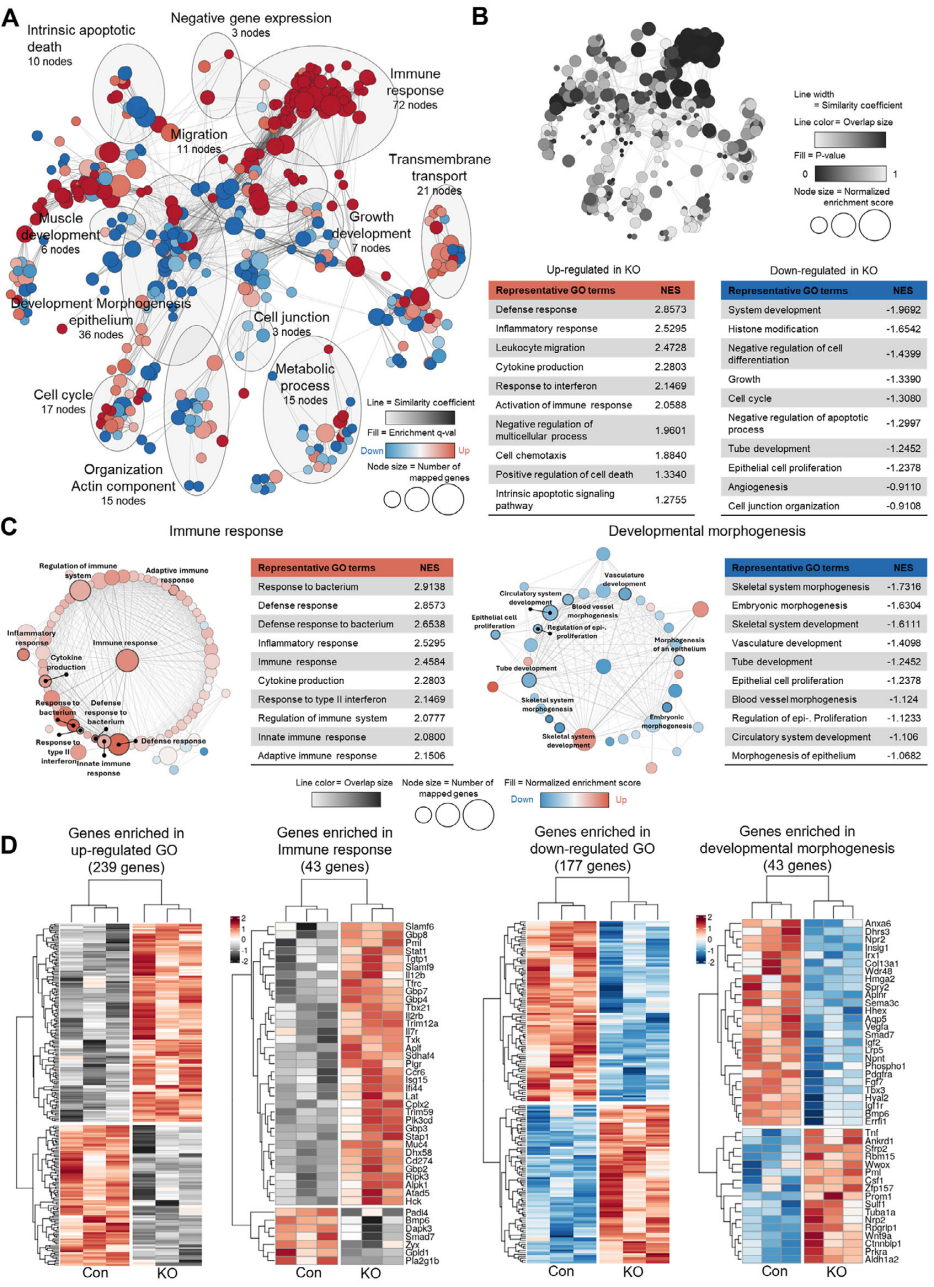
Two‐week deletion of PRMT1 in ECs alters the expression of genes related to immune response and developmental morphogenesis in lungs. A) Transcriptome analysis of lungs from Con and KO mice (*n* = 3). GO Biological Process annotation for the entire coexpression network. Line, similarity coefficient. Filled color, enrichment *q*‐value. Node size, number of mapped genes. B) A black and white summary version of GO annotation (upper panel). GO analysis (lower panel) of upregulated genes (left) and downregulated genes (right) in KO mice. Line, similarity coefficient. Line color, overlap size. Fill, *p*‐value. Node size, normalized enrichment score. C) Representative GO terms associated with genes involved in immune response (left) and developmental morphogenesis processes (right). Line color, overlap size. Node size, number of mapped genes. Fill, normalized enrichment score. D) Heatmap visualization of up‐ and downregulated gene in KO versus Con lungs (*n* = 3), particularly in immune response and developmental morphogenesis gene enrichment.

To determine the underlying molecular mechanism responsible for the pulmonary phenotype of KO lungs, we conducted bulk RNA sequencing analysis using total lung RNA from Con and KO mice. The subsequent transcriptome analysis utilizing the total lung RNAs revealed the differential expression of ≈725 genes between Con and KO lungs. The gene ontology (GO) network analysis indicated that genes related to immune response (72 nodes) and developmental morphogenesis (36 nodes) were mainly up‐ and downregulated in KO lungs, respectively (**Figure** **3**A). Among the 392 upregulated and 333 downregulated genes in KO lungs, the upregulated genes were predominantly associated with defense response, inflammatory response, leukocyte migration, cytokine production, response to interferon, activation of immune response, and others. Conversely, the downregulated genes were mainly linked to system development, histone modification, negative regulation of cell differentiation, growth, and cell cycle (Figure 3B). Detailed gene association analysis revealed a concurrent upregulation of genes related to the immune response and a downregulation of genes associated with developmental morphogenesis (Figure 3C). Heatmap analysis of genes enriched in the immune response indicated the upregulation of genes involved in interferon and interleukin pathways, including *Ifi‐44*, *Isg‐15*, *Il‐7r*, *Il‐12b*, *Il‐2rb*, *Trim12a*, *Trim59*, *Gbp8*, *Gbp4*, and *Stat1* (Figure 3D). These findings suggest an activated inflammatory response in KO lungs. Conversely, genes associated with growth factor signaling and regulation of EC proliferation, such as *Vegf‐a*, *Igf‐2*, *Igf‐1r*, *Fgf‐7*, *Pdgfr‐α*, *Bmp‐6*, *Lrp‐5*, and *Aplnr*, were downregulated in KO lungs. Collectively, these findings suggest that endothelial PRMT1 ablation leads to alterations in the expression of genes associated with immune/inflammatory response and developmental morphogenesis within the lung tissue.

### 2.4 Single Cell Sequencing Reveals That PRMT1 Ablation Shifts Capillary ECs toward an Inflammatory State

**Figure 4:**
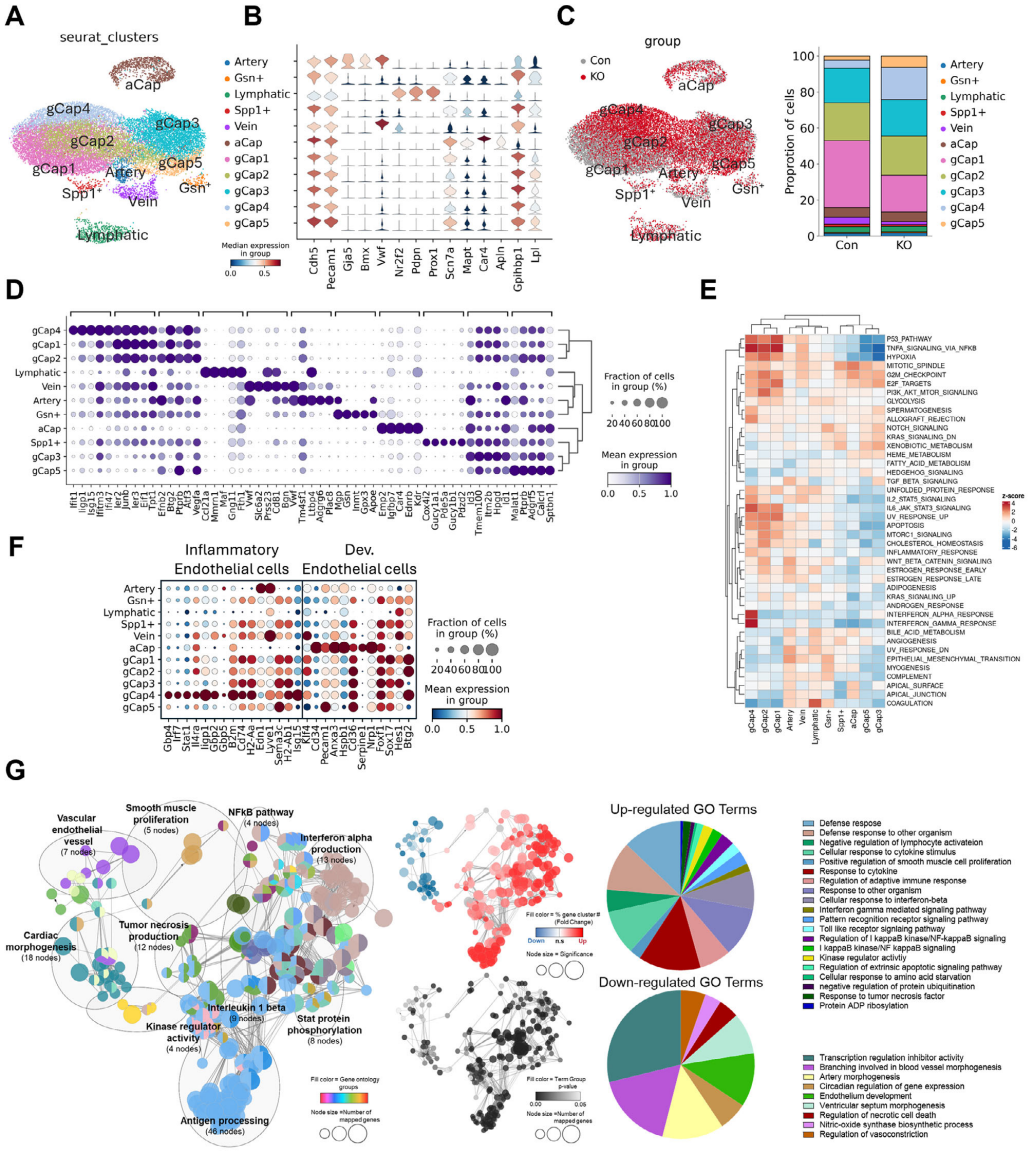
Single cell RNA sequencing analysis reveals upregulation of immune response‐related signaling in pulmonary endothelial subpopulations. A) UMAP visualization depicting pulmonary EC subpopulations in Con (left) and KO (right) mice. B) Violin plot of marker genes in artery, vein, lymphatics, and capillary ECs with common endothelial genes. C) UMAP plot of pulmonary ECs in lungs from Con and KO mice (left). The bar graph displays the proportion of pulmonary EC subpopulations in Con and KO mice (right). D) Dot plots illustrate top ranked genes for pulmonary endothelial clusters. Color, average expression. Dot size, percent expression. E) Heatmap of GSEA based hallmark gene ontology analysis in pulmonary ECs. F) Dot plots visualize gene sets involved in the inflammation response (left) and developmental processes (right) of endothelial clusters. Color, average expression. Dot size, percent expression. G) Network plot depicting gene ontology analysis. Color, gene ontology groups. Node size, number of mapped genes. Red and blue summary version of GO annotation (upper panel). Fill color, fold change. Node size, significant level. Black and white summary version of GO annotation (lower panel). Fill color, *p*‐value. Node size, number of mapped genes. Pie charts displaying upregulated (upper panel) and downregulated (lower panel) GO terms in ECs of KO mouse.

To explore gene expression changes in ECs from the lungs of Con and KO mice, we conducted single cell sequencing analysis with CD45‐negative/CD31‐positive ECs isolated from Con and KO lungs (Figure S7, Supporting Information). Through unsupervised clustering, 11 major cell clusters from a total of 11 457 cells from KO lungs and 8249 cells from Con lungs were manually annotated and visualized using UMAP dimensionality reduction in integrated Con and KO samples (**Figure** **4**A). The substantiation of 11 clusters as ECs was established through the robust expression of well‐recognized EC markers, including *Cdh5* and *Pecam1* (Figure S8A,B, Supporting Information). ECs typically exhibit a distinctive classification into five subclusters, each characterized by specific markers associated with arterial (*Gja5*, *Bmx*), venous (*Vwf*, *Nr2f2*), lymphatic (*Pdpn*, *Prox1*), general (*Gpihbp1*, *Lpl*), and aerocyte (*Car4*, *Apln*) (Figure 4B and Figure S8C, Supporting Information). The last two are subtypes of capillaries (*Scn7a*, *Mapt*), commonly denoted as gCap and aCap, respectively. The gCap populations were further grouped into five clusters based on the differential expression profile of markers.

Gene rank analysis revealed a clear cellular distinction among the EC clusters; however, no substantial overall differences were observed between Con and KO ECs (Figure S8D, Supporting Information). A notable exception was observed within the gCap clusters, where the KO sample exhibited an increased proportion of gCap4 cluster and a decreased proportion of gCap1 cluster compared to the Con sample (Figure 4C). Among the five gCap subpopulations, gCap4 demonstrated the highest upregulation of genes associated with interferon/immune response, including *Ifit‐1*, *Iigp‐1*, interferon‐stimulated gene 15 (*Isg‐15)*, *Ifitm‐3*, and *Ifi‐47* (Figure 4D). Subsequent GO analysis revealed a strong association of Interferon response and TNF‐α signaling with gCap4 (Figure 4E). To further characterize the gCap4 subset, we utilized a previously identified gene set for inflammatory and developmental ECs^[^ ^25^ ^]^ to analyze gene expression patterns. Our data demonstrated that gCap4 exhibited an upregulation of genes associated with inflammatory ECs (Figure 4F). Additionally, we validated the upregulation of inflammatory markers, including *ISG* *‐15*, *STAT1*, and *IRF‐7*, in furamidine‐treated HUVECs (Figure S9, Supporting Information).

To delve into the gCap4 properties, the enrichment analysis based on differentially expressed genes (DEGs) in the gCap4 cluster was performed. Gene set enrichment analysis (GSEA) unveiled a notable upregulation in genes associated with diverse biological processes, including antigen processing (46 nodes), interferon alpha production (13 nodes), tumor necrosis factor production (12 nodes), interleukin 1 beta regulation (9 nodes), stat protein phosphorylation (8 nodes), smooth muscle proliferation (5 nodes), kinase regulator activity (4 nodes), and NF‐κB pathway activation (4 nodes). Conversely, genes implicated in cardiac morphogenesis (18 nodes) and vascular endothelial vessel development (7 nodes) were downregulated (Figure 4G). These findings led us to hypothesize that endothelial dysfunction triggered by PRMT1 ablation is manifested by the increase in inflammatory gCap4 cluster‐associated ECs.

Utilizing a single‐cell fate analysis algorithm implemented in scFates, we conducted a comprehensive investigation into the five distinct subpopulations within the gCap population. Employing the ElPiGraph algorithm, we constructed a Force Atlas layout‐based UMAP dimensionality reduction plot featuring a curve delineated by 120 nodes of the gCap populations (Figure S10A, Supporting Information). Node 17, situated within the gCap1 subpopulation, was selected as the root to generate a trajectory of the ECs (Figure S10B, Supporting Information). The analysis revealed a trajectory from gCap1 to gCap4. Along the trajectory, the transition of gene expression pattern from developmental genes to immune response‐related genes was observed (Figure S10C, Supporting Information). Additional heatmaps illustrated the transitions from the homeotic state of gCap1 to other cell states (gCap2, gCap3, gCap4, and gCap5) (Figure S10D, Supporting Information). Dendrograms of inflammation‐related genes, such as *Isg‐15*, *Ripk‐1*, and *NFκbiz*, showed increased expression during the transition from gCap1 to gCap4 (Figure S10E, Supporting Information). These findings collectively suggest that the ablation of PRMT1 in ECs induces a dynamic transformation of capillary ECs to an inflammatory state in the lung tissue.

### 2.5 PRMT1 Regulates p65/NF‐κB Activity via Asymmetric Dimethylation in ECs

**Figure 5:**
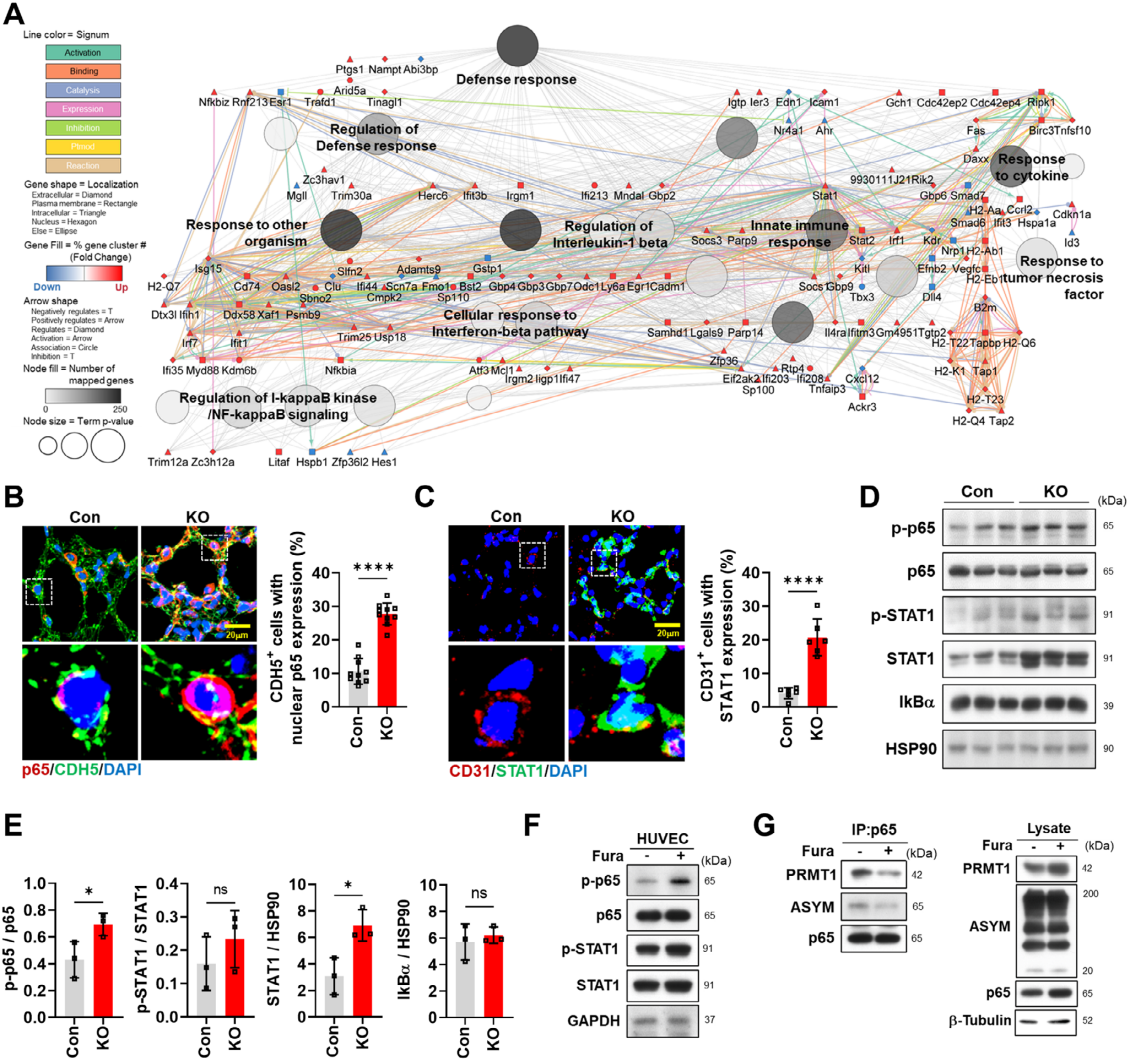
PRMT1 ablation results in inflammatory responses through NF‐κB hyperactivation. A) Gene and pathway interaction networks in “defense response” visualized using ClueGO/CluePedia plugin from Cytoscape. The connectivity of the pathway in the network is described by functional nodes and edges that are shared between the DEGs with kappa score of 0.4. The enrichment shows only significant pathway (*p* < 0.05). The value of *p* < 0.05 indicates the node size. The color represents various molecular pathways involved in the enrichment analysis of the identified DEGs. B) Confocal images of alveolar region stained for p65 (red), CDH5 (green), counterstained with DAPI (blue) in Con or KO lung. Scale bar = 20 µm. Quantification of the percentage of CDH5‐positive cells with nuclear p65 expression. C) Confocal images of alveolar region stained for CD31 (red), STAT1 (green) and counterstained with DAPI (blue) in Con or KO lung. Scale bar = 20 µm. Quantification of the percentage of CD31‐positive cells with STAT1 expression. D) Immunoblot analysis of p‐p65, p65, p‐STAT1, STAT1, IκBα, and loading control (HSP90) protein with lung lysates of Con and KO mice (*n* = 3). E) Quantification of relative protein levels of p‐p65 to p65, p‐STAT1 to STAT1, STAT1 and IκBα to HSP90. F) Immunoblot analysis of p‐p65, p65, p‐STAT1, STAT1, and loading control (GAPDH) in HUVECs treated with dimethyl sulfoxide (DMSO) or furamidine (Fura, 20 µm) for 2 h. G) Analysis of asymmetric methylation patterns on the p65 subunit of NF‐κB in DMSO or Fura‐treated HUVECs. Cellular lysates were subjected to immunoprecipitation, followed by immunoblotting using an anti‐p65 antibody. Data for all panels are means ± SD. Student's *t*‐test. ns = not significant, *p < 0.05, ****p < 0.0001.

To investigate whether the upregulation of interferon/immune response in gCap4 was influenced by external factors, a ligand‐receptor interaction analysis was conducted (Figure S11, Supporting Information). The analysis did not reveal any significant interactions involving secreted factors, interactive factors, or extracellular matrix (ECM) proteins specifically associated with gCap4 cluster, suggesting an intrinsic mechanism underlying the transition of gCap subpopulations. Next, the interaction network analysis of the top upregulated genes in gCap4 subset revealed a crosstalk of NF‐κB signaling with defense and immune responses (**Figure** **5**A). Based on these findings, we explored the PRMT1‐mediated regulatory dynamics involving NF‐κB in pulmonary ECs.

The immunostaining for p65 and STAT1 showed a substantial increase in the number of ECs exhibiting nuclear localization of p65/NF‐κB and the expression of STAT1 in KO lungs, whereas control ECs showed predominantly cytoplasmic p65/NF‐κB localization and very low STAT1 staining (Figure 5B,C). KO lungs exhibited elevated levels of phosphorylated p65/NF‐κB (p‐p65) and STAT1 proteins while p‐STAT1 and IκBα proteins did not show any significant changes (Figure 5D,E). To verify the role of PRMT1 in the regulation of p65/NF‐κB in ECs, HUVECs were treated with a PRMT1 inhibitor furamidine for 2 h, followed by immunoblot analysis (Figure 5F). Furamidine treatment resulted in an upregulation of p‐p65 and p‐STAT1 proteins in HUVECs. Although furamidine treatment elevated the level of PRMT1 and p65 proteins in total lysates, less PRMT1 proteins were immunoprecipitated with anti‐p65 antibodies, correlated with reduced ASYM‐detected p65 protein (Figure 5G), suggesting for the requirement of the PRMT1 activity for its p65 interaction. These data suggest that PRMT1 might regulate pulmonary EC function via controlling NF‐κB signaling.

### 2.6 Mice with Heterozygous KO for Endothelial PRMT1 Exhibit Aggravated Alveolar Destruction, Cell Death, and Inflammation in Lungs of PPE‐Triggered COPD Model

**Figure 6:**
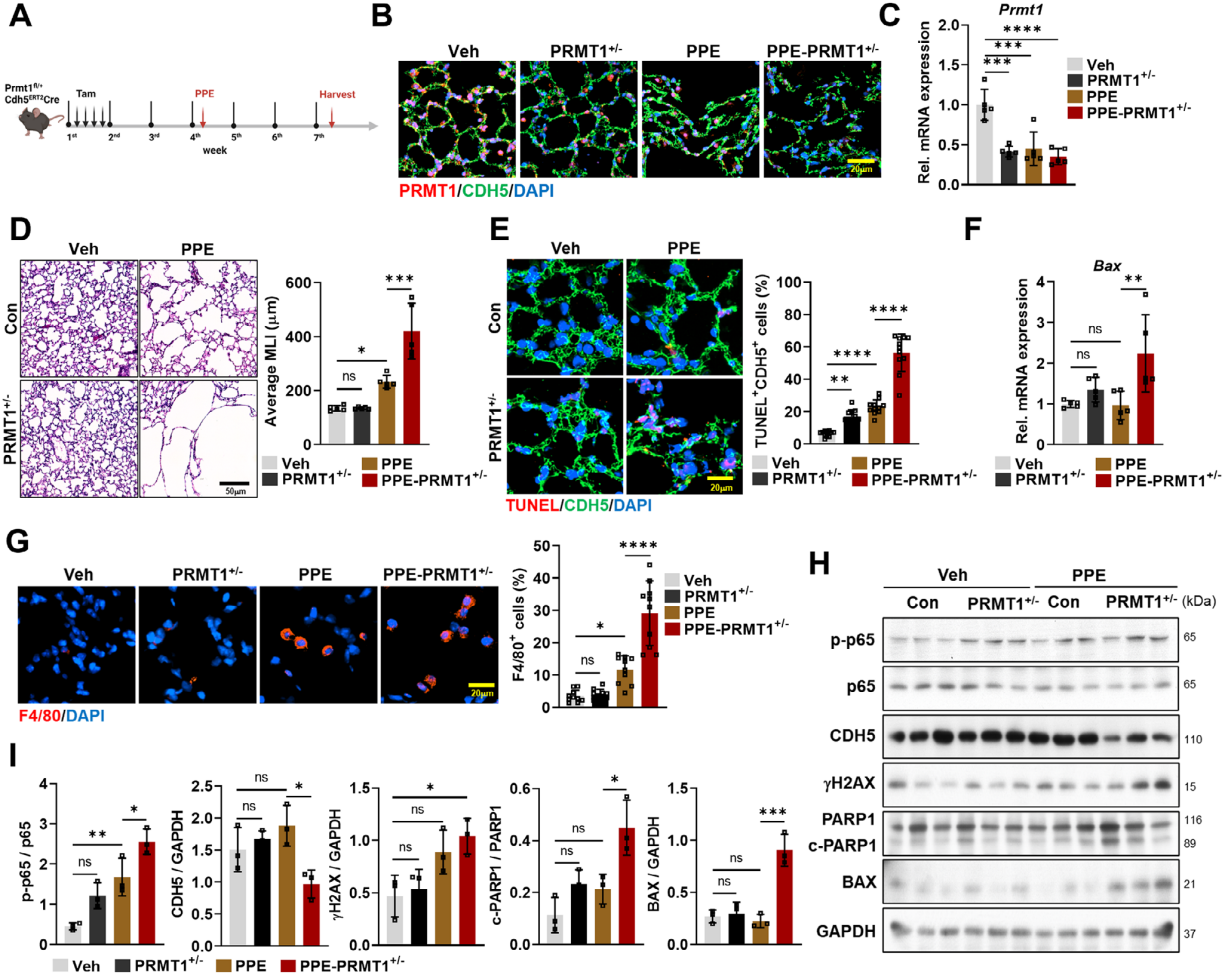
Endothelial PRMT1 heterozygous KO mice exhibit severe COPD phenotypes, associated with increased alveolar destruction, cell death, and inflammation. A) Scheme of tamoxifen and PPE injection for generating heterozygous endothelial PRMT1 KO with COPD mouse model. B) Confocal images of alveolar area, stained for PRMT1 (red), CDH5 (green), and counterstained with DAPI (blue). Scale bar = 20 µm. C) Quantitative reverse transcription polymerase chain reaction analysis of *Prmt1* expression using total lung mRNA from vehicle (Veh), PRMT1^+/−^, PPE treatment (PPE), and PPE treated PRMT1^+/−^(PPE‐PRMT1^+/−^) mice (*n* = 5). D) Histological analysis of lungs from Veh, PRMT1^+/−^, PPE, and PPE‐PRMT1^+/−^mice using H&E staining. Scale bar = 50 µm. Quantification of MLI alveolar regions. E) Confocal images of TUNEL assay of alveolar area, stained for cell death (TUNEL positive, red), CDH5 (green), and counterstained with DAPI (blue). Scale bar = 20 µm. Quantification of percentage of CDH5‐positive TUNEL‐positive cells in alveolar area. F) qRT‐PCT analysis of apoptosis maker *Bax* using total lung mRNA. G) Confocal images of alveolar area, stained for macrophage marker F4/80 (red), and counterstained with DAPI (blue). Scale bar = 20 µm. Quantification of F4/80 positive cells in the alveolar area. H) Immunoblot analysis for protein expression levels of p‐p65, p65, CDH5, γH2AX, PARP1, cleaved PARP1 (c‐PARP1), BAX, and loading control (GAPDH) using total lung lysates from Veh, PRMT1^+/−^, PPE, and PPE‐PRMT1^+/−^mice (n = 3). I) Quantification of relative protein level of p‐p65 to p65, c‐PARP1 to PARP1, CDH5, γH2AX, and BAX to GAPDH. Data for all panels are means ±SD. One‐way analysis of variance (ANOVA). ns = not significant, *p < 0.05, **p < 0.01, ***p < 0.001, ****p < 0.0001.

Next, we investigated the effect of the declined PRMT1 expression in ECs on COPD phenotype using tamoxifen‐inducible endothelial PRMT1 heterozygous KO mice (*Prmt1* ^fl/+^; *Cdh5* ^ERT2–Cre^). Two weeks after the final vehicle or tamoxifen injection, mice were administered a single nasal dose of phosphate‐buffered saline (PBS) or PPE to induce COPD‐like phenotype (**Figure** **6**A). PRMT1 levels were significantly reduced in PPE‐treated mice and further diminished in PPE‐treated PRMT1^+/−^ mice (Figure 6B,C). Histological analysis revealed that PPE‐treated PRMT1^+/−^ lungs exhibited markedly enlarged alveolar spaces, compared to both vehicle‐and PPE‐treated control mice (Figure 6D). In addition, the expression of *Bax* transcript and the proportion of TUNEL‐positive CDH5^+^ cells were substantially increased in PPE‐treated PRMT1^+/−^ mice (Figure 6E,F). PPE‐treated lungs showed heightened macrophage infiltration, compared to PPE‐treated control lungs (Figure 6G). However, a further increase in macrophage recruitment was observed in PPE‐treated PRMT1^+/−^ lungs. In addition, fibrosis was substantially enhanced in the alveolar region of PPE‐treated PRMT1^+/−^ mice, compared to PPE‐treated control mice (Figure S12A,B, Supporting Information). These findings propose a crucial role of PRMT1 in the progression of COPD.

To delve into the relationship between PRMT1 and p65/NF‐κB in COPD, we examined p65/NF‐κB and cellular damage signaling. The level of p‐p65 was significantly increased in PPE‐treated control lungs and further increased in PPE‐treated PRMT1^+/−^ mice (Figure 6H,I). While CDH5 distinctively declined in PPE‐treated PRMT1^+/−^ lungs, PPE‐treated control lungs showed no significant changes. Furthermore, PPE‐treated PRMT1^+/−^ lungs exhibited aggravated cell death evident by enhanced expression of γH2AX and cell death markers c‐PARP1 and BAX proteins compared to PPE‐treated control lungs. Coimmunostaining of γH2AX and CD31 confirmed a marked increase in DNA damage in pulmonary ECs of PPE‐treated PRMT1^+/−^ lungs (Figure S12C, Supporting Information). These findings further support that dysregulation of NF‐κB signaling is attributable to the COPD pathogenesis in PRMT1^+/−^ mice.

### 2.7 Ablation of PRMT1 Causes Endothelial Senescence in COPD Lungs and TNF‐α‐Triggered Endothelial Dysfunction

**Figure 7:**
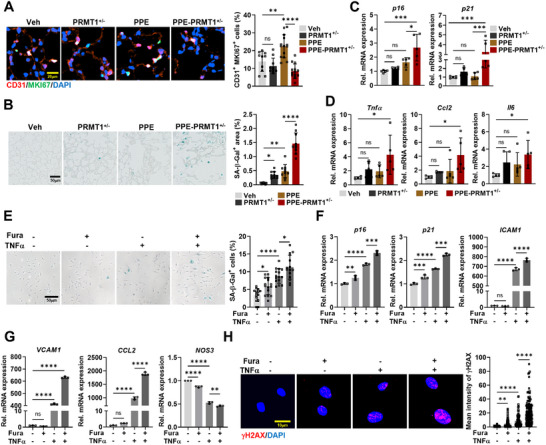
Inhibition of PRMT1 causes endothelial senescence in COPD lungs and TNF‐α‐induced endothelial dysfunction. A) Confocal images of lung tissues, CD31 (red), proliferation marker (MKI67, green), and counterstained with DAPI (blue). Scale bar = 20 µm. Quantification of proliferating ECs (CD31^+^MKI67^+^) of the lung tissues from Veh, PRMT1^+/−^, PPE, and PPE‐PRMT1^+/−^mice. B) Photographs of lung tissue stained for senescence associated β‐Galactosidase (SA‐β‐Gal). Scale bar = 50 µm. Quantification of the percentage of SA‐β‐Gal positive in alveolar area of Veh, PRMT1^+/−^, PPE, and PPE‐PRMT1^+/−^mice. C,D) qRT‐PCR analysis of *p16*, *p21*, *Tnf‐α*, *Ccl‐2*, and *Il‐6* expression levels using total lung mRNA from Veh, PRMT1^+/−^, PPE, and PPE‐PRMT1^+/−^mice (*n* = 5). E) Photographs of SA‐β‐Gal staining of HULECs treated with DMSO, Fura (20 µm), TNF‐α (50 ng mL^−1^), or a combination of Fura and TNF‐α for 24 h. Scale bar = 50 µm. Quantification of SA‐β‐Gal‐positive HULECs. F,G) qRT‐PCR analysis of *p16*, *p21*, *ICAM‐1*, *VCAM‐1*, *CCL‐2*, and *NOS3* mRNA expression in HULECs treated with the same condition in (E). H) Confocal images of HULECs, stained for DNA damage (γH2AX, red), and counterstained with DAPI (blue). Scale bar = 10 µm. Quantification of mean intensity of γH2AX in HULECs treated with the same condition in (E). Data for all panels are means ±SD. One‐way ANOVA. ns = not significant, *p < 0.05, **p < 0.01, ***p < 0.001, ****p < 0.0001.

In addition to endothelial homeostasis, proSP‐C‐positive alveolar type II cells are activated upon injury and differentiate into alveolar type I cells, important for lung regeneration and homeostasis.^[^ ^26^ ^]^ Thus, we investigated the number of ECs and alveolar cells by immunostaining for the EC marker, CD31, and the alveolar type II cell marker, proSP‐C. Interestingly, the percentage of CD31‐positive cells was significantly reduced, while the number of proSP‐C positive alveolar cells was markedly increased in the alveolar areas of PPE‐treated mice, with a more pronounced changes observed in PPE‐treated PRMT1^+/−^ mice (Figure S13, Supporting Information). EC proliferation was assessed by coimmunostaining for CD31 and MKI67 (**Figure** **7**A). PPE‐treated lungs exhibited a notable increase in CD31^+^/MKI67^+^ cell proportion while such increase was significantly abrogated in PPE‐treated PRMT1^+/−^ lungs. These findings suggest that PRMT1 plays a critical role in EC proliferation in response to lung injury and its partial deficiency affects alveolar homeostasis and regeneration.

Impaired cell proliferation with increased inflammatory response is a hallmark feature of cell senescence associated with COPD pathogenesis.^[^ ^27^ ^]^ The lung tissues of PPE‐treated PRMT1^+/−^ mice exhibited upregulated expression of senescence‐related genes, including *p16*, *p21*, *Tnf‐α*, *Ccl‐2*, and *Il‐6*, as well as increased activity of senescence‐associated β‐galactosidase (SA‐β‐Gal; Figure 7B–D). We further investigated the effect of PRMT1 inhibition on TNF‐α‐triggered senescence in Human lung endothelial cells (HULECs). Furamidine‐treated HULECs exhibited a mild but significant increase in SA‐β‐Gal‐positive cells that were further increased upon TNF‐α treatment (Figure 7E). Consistently, TNF‐α treatment markedly increased the transcript and protein levels of senescence markers p16 and p21 and the transcript of other markers such as intercellular adhesion molecule 1(*ICAM* *‐1)*, vascular cell adhesion molecule 1 (*VCAM* *‐1)*, and C‐C motif chemokine ligand 2 (*CCL* *‐2)* mRNAs that were further increased by furamidine treatment (Figure S14A, Supporting Information, and Figure 7F,G). Conversely, *NOS3* expression was notably decreased following furamidine treatment alone and further declined with TNF‐α treatment. Additionally, furamidine treatment of HULECs elicited a mild but significant increase in γH2AX^+^ cells that were markedly elevated by TNF‐α treatment (Figure 7H). Similar results were also obtained with HUVECs treated with furamidine and TNF‐α (Figure S14B, Supporting Information). Furamidine‐treated cells exacerbated oxidative stress and DNA damage in response to TNF‐α compared to vehicle‐treated cells. Additionally, furamidine treatment significantly enhanced the TNF‐α‐triggered response, as evidenced by the notable increase in inflammatory genes such as *ICAM‐1*, *VCAM‐1*, *ISG‐15*, *IL‐6*, *IL‐1α*, *IL‐8*, *CCL‐2*, *NOX4*, and *EDN1*, compared to vehicle‐treated HUVECs (Figure S14C,D, Supporting Information). In contrast, *NOS3* was significantly declined by furamidine treatment and further reduced by TNF‐α treatment. Consistently, furamidine treatment reduced the percentage of BrdU‐positive cells and substantially decreased *MKI67* expression (Figure S14E,F, Supporting Information). Taken together, these data suggest that PRMT1 is required for suppression of endothelial dysfunction and senescence induced by TNF‐α.

### 2.8 Inhibition of NF‐κB Abrogates TNF‐α‐ and Furamidine‐Induced EC Dysfunction

**Figure 8:**
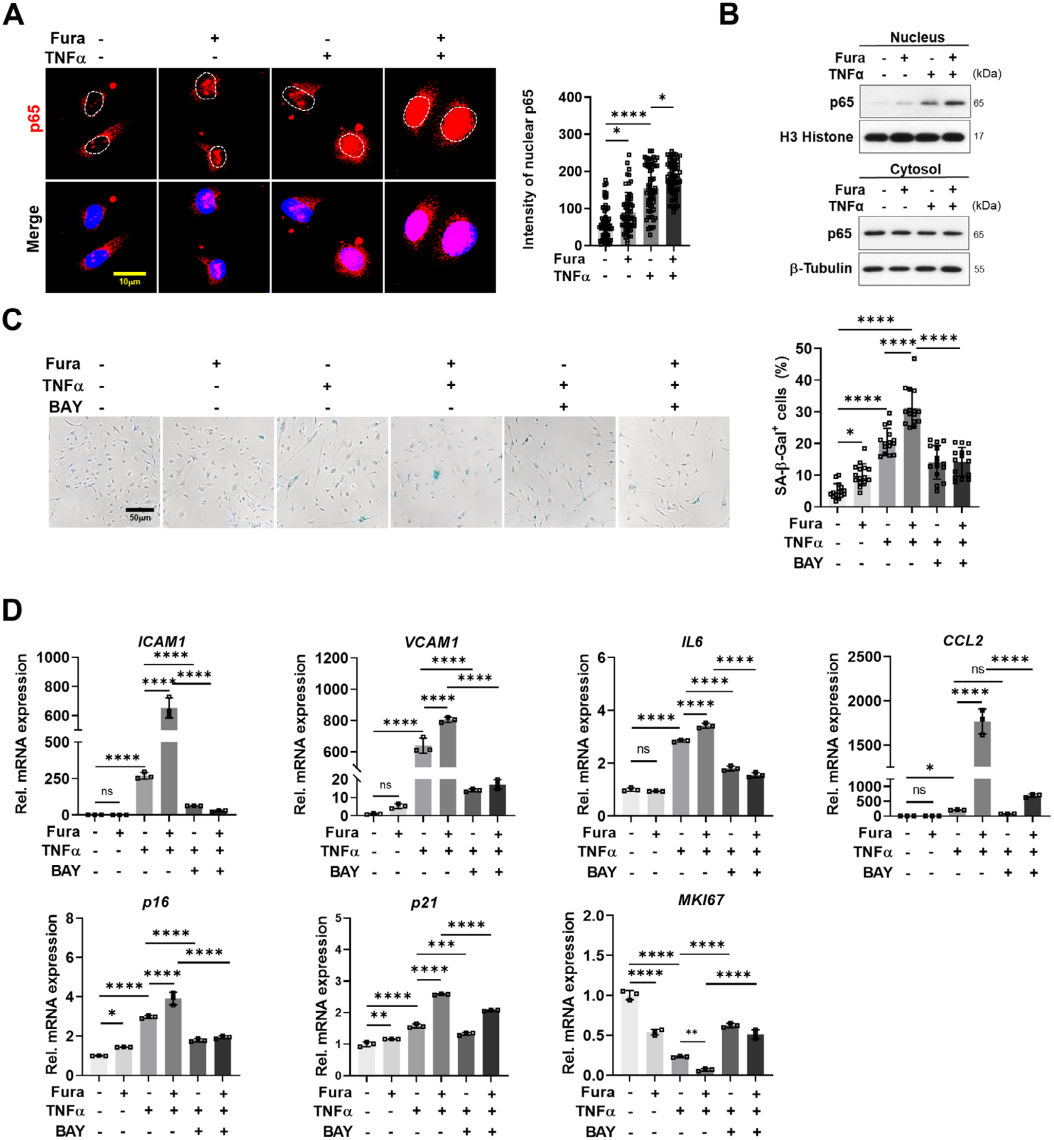
NF‐κB signaling inhibition by BAY‐11‐7082 rescues TNF‐α‐induced endothelial dysfunction. A) Confocal images of HULECs treated with DMSO, Fura (20 µm), TNF‐α (50 ng mL^−1^), or a combination of Fura and TNF‐α, stained for p65 (red) and counterstained with DAPI (blue). Scale bar = 10 µm. Quantification of mean intensity of nucleus localized p65 in HULECs. B) Immunoblot analysis of p65 and loading controls (Histone H3 for nucleus and β‐Tubulin for cytosol) protein levels using nuclear and cytosolic lysates from HULECs. C) Photograph of SA‐β‐Gal staining in HULECs treated with DMSO, Fura (20 µm), TNF‐α (50 ng mL^−1^), BAY‐11‐7082 (2 µm) and TNF‐α, or a combination of Fura, BAY‐11‐7082, and TNF‐α for 24 h. Scale bar = 50 µm. Quantification of SA‐β‐Gal positive HULECs. D) qRT‐PCR analysis of *ICAM‐1*, *VCAM‐1*, *IL‐6*, *CCL‐2*, *p16*, *p21*, and *MKI67* mRNA level of HULECs treated with the same conditions in (C). Data for all panels are means ± SD. One‐way ANOVA. ns = not significant, *p < 0.05, **p < 0.01, ***p < 0.001, ****p < 0.0001.

To gain insights into the importance of NF‐κB signaling in endothelial dysfunction triggered by PRMT1 inhibition, we examined the nuclear localization of p65/NF‐κB in response to furamidine and TNF‐α treatment by immunostaining and nuclear fractionation (**Figure** **8**A,B). Control‐treated HULECs exhibited a predominant cytoplasmic localization of p65 proteins while furamidine treatment elicited a nuclear trans‐localization of p65 that were further enhanced by TNF‐α treatment, likely reflecting the active states of p65/NF‐κB proteins. To evaluate the significance of p65/NF‐κB activity in cellular senescence triggered by PRMT1 inhibition and TNF‐α, HULECs were treated with an NF‐κB inhibitor BAY‐11‐7082 in combination of TNF‐α and furamidine. BAY‐11‐7082 treatment attenuated the increase in SA‐β‐Gal‐positive cells elicited by TNF‐α and furamidine treatment (Figure 8C). Furthermore, the treatment with BAY‐11‐7082 significantly reduced the expression of senescence markers, including *ICAM‐1*, *VCAM‐1*, *IL‐6*, *CCL‐2*, *p16*, and *p21* in TNF‐α‐treated and furamidine plus TNF‐α‐treated HULECs, while *MKI67* expression was restored (Figure 8D). These findings indicate that NF‐κB signaling is implicated in endothelial senescence induced by TNF‐α. Collectively, our current study demonstrates a crucial role for PRMT1 in pulmonary endothelial function via controlling p65/NF‐κB signaling to suppress inflammation, critical for the pathogenesis of COPD.

### 2.9 Endothelial PRMT1 Overexpression Mitigates TNF‐α‐Induced Endothelial Dysfunction and Restores Pulmonary Function in a COPD Model

**Figure 9:**
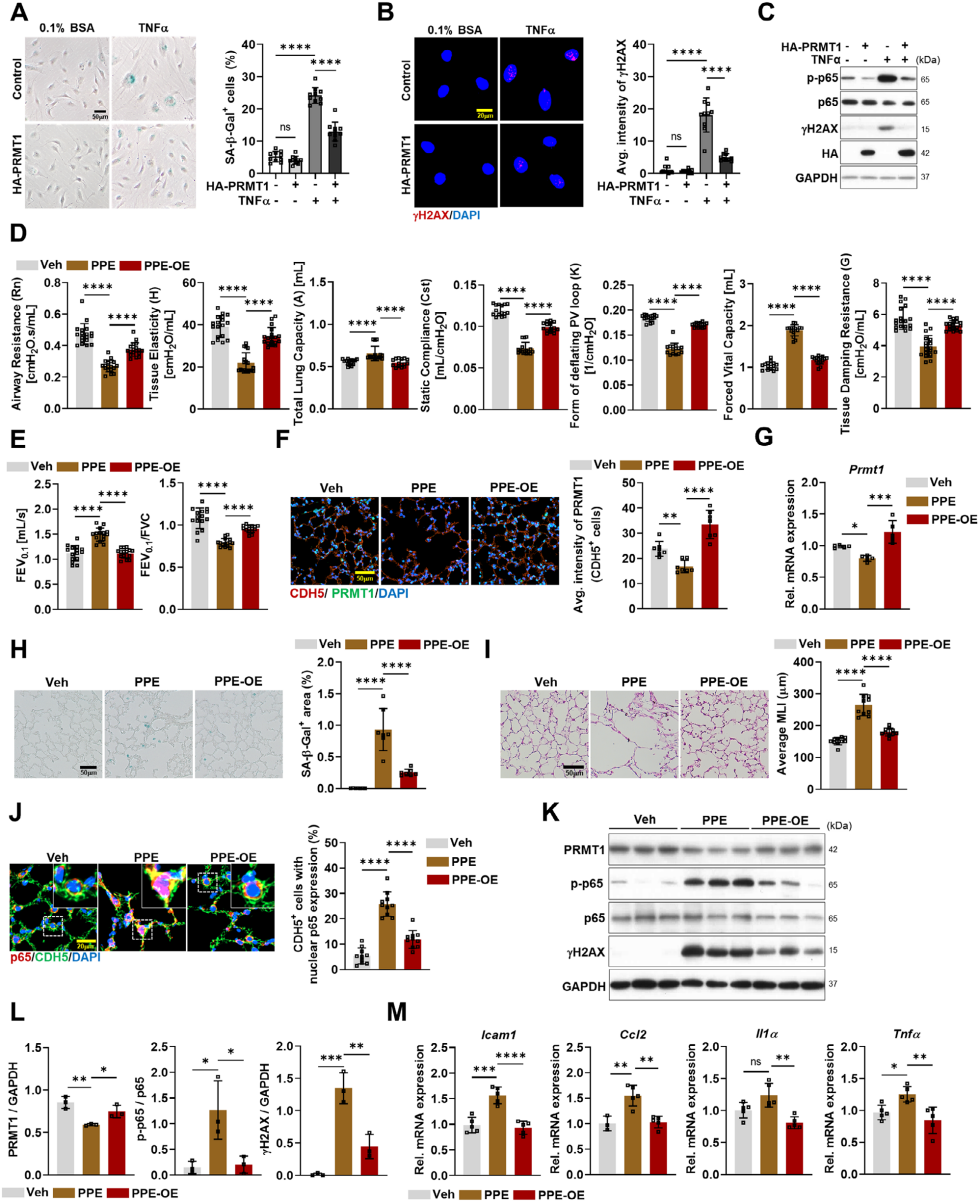
PRMT1 overexpression attenuates TNF‐α‐triggered endothelial damage and PPE‐induced pulmonary dysfunction. A) Photographs of SA‐β‐Gal staining of HULECs transfected with pcDNA3.1 vector (Control) or pcDNA3.1‐HA‐PRMT1 (HA‐PRMT1) for 24 h, followed by treatment of 0.1% bovine serum albumin (BSA) or TNF‐α (50 ng mL^−1^) for 24 h. Scale bar = 50 µm. Quantification of SA‐β‐Gal‐positive HULECs. B) Confocal images of HULECs, stained for DNA damage (γH2AX, red) and counterstained with DAPI (blue). Scale bar = 20 µm. Quantification of mean intensity of γH2AX in HULECs treated with the same condition in (A). C) Immunoblot analysis of p‐p65, p65, γH2AX, HA, and GAPDH protein levels of HULECs treated with the same condition in (A). D,E) Respiratory function assessment of vehicle (Veh), PPE treated (PPE), or endothelial PRMT1 overexpression with PPE‐treated mice (PPE‐OE) based on various parameters, including airway resistance (Rn, cmH₂O·s mL^−1^), tissue elasticity (H, cmH₂O mL^−1^), total lung capacity (A, mL), static compliance (Cst, mL cmH₂O^−1^), form of deflating PV loop (K, 1 cmH₂O^−1^), force vital capacity (FVC, mL), tissue damping resistance (G, cmH₂O mL^−1^), forced expiratory flow at 0.1 s (FEV_0.1_, mL s^−1^), and FEV_0.1_/FVC ratio. F) Confocal images of alveolar area, stained for CDH5 (red), PRMT1 (green), and counterstained with DAPI (blue). Scale bar = 50 µm. Quantification of average intensity of PRMT1 in CDH5 positive cells in Veh, PPE, or PPE‐OE mice. G) qRT‐PCR analysis of *Prmt1* using total lung RNA from Veh, PPE, or PPE‐OE mice (*n* = 5). H) Photographs of lung tissue stained for SA‐β‐Gal. Scale bar = 50 µm. Quantification of the percentage of SA‐β‐Gal positive in alveolar area of Veh, PPE, and PPE‐OE mice. I) Histological analysis of lungs from Veh, PPE, or PPE‐OE using H&E staining. Scale bar = 50 µm. Quantification of MLI alveolar regions. J) Confocal images of alveolar area, stained for p65 (red), CDH5 (green), and counterstained with DAPI (blue). Scale bar = 20 µm. Quantification of the percentage of CDH5‐positive cells with nuclear p65 expression in Veh, PPE, or PPE‐OE mice. K) Immunoblot analysis for protein expression levels of PRMT1, p‐p65, p65, γH2AX, and loading control (GAPDH) using total lung lysates from Veh, PPE, and PPE‐OE mice (*n* = 3). L) Quantification of relative protein level of p‐p65 to p65, PRMT1, and γH2AX to GAPDH. M) qRT‐PCR analysis of *Icam‐1*, *Ccl‐2*, *Il‐1α*, and *Tnf‐α* using total lung RNA from Veh, PPE, or PPE‐OE mice (*n* = 5). Data for all panels are means ± SD. One‐way ANOVA. ns = not significant, *p < 0.05, **p < 0.01, ***p < 0.001, ****p < 0.0001.

To assess the protective role of PRMT1 in ECs under inflammatory conditions, HULECs were transfected with either pcDNA3.1 vector (Control) or pcDNA3.1‐Hemagglutinin‐PRMT1 (HA‐PRMT1) and subsequently treated with TNF‐α (50 ng mL^−1^) for 24 h. PRMT1 overexpression significantly reduced the percentage of SA‐β‐Gal‐positive cells and attenuated the TNF‐α‐induced increase in γH2AX expression (**Figure** **9**A,B). Moreover, PRMT1‐overexpressing HULECs displayed suppressed levels of phosphorylated p65 (p‐p65) under both basal and TNF‐α treatment conditions (Figure 9C). This overexpression also mitigated the mRNA expression of inflammatory markers, such as *ICAM‐1*, *VCAM‐1*, and *CCL‐2*, as well as senescence markers, including *p16* and *p21* (Figure S15, Supporting Information).

To verify the in vivo effects of PRMT1, a COPD model was employed. Mice were intravenously injected with an AAV2‐QUADYF virus encoding the *Prmt1* gene under the control of EC‐specific *Cd144* promoter, allowing targeted PRMT1 overexpression in ECs. Following virus delivery, mice were treated with PPE to induce COPD over a two‐week period (Figure S16A, Supporting Information). Respiratory function in these mice was assessed using the FlexiVent FX system (SCIREQ Inc., Montreal, Canada) (Figure 9D,E). Results showed that PPE‐induced airway resistance, tissue damping resistance, and tissue elasticity were significantly alleviated in PRMT1 overexpressing mice (PPE‐OE). Furthermore, while PPE treatment increased total lung capacity (TLC) due to alveolar destruction and airspace enlargement, PRMT1 overexpression reversed this effect, reducing TLC. Static compliance and the deflation pattern of the pressure‐volume (PV) loop were also improved in PPE‐OE mice. Forced vital capacity (FVC) and forced expiratory flow at 0.1 s (FEV_0.1_) decreased, leading to a higher FEV_0.1_/FVC ratio. These findings demonstrate that PRMT1 overexpression improves respiratory function in the COPD mouse model.

Increased PRMT1 gene expression and protein levels in the lungs, specifically in CDH5^+^ pulmonary cells, confirmed successful PRMT1 overexpression (Figure 9F,G). In PPE‐OE mice, alveolar enlargement, SA‐β‐Gal expression, and the levels of senescence markers, such as *p16* and *p21*, were reduced, without affecting lung or other tissues weights (Figure 9H,I and Figure S16C, Supporting Information).

Consistent with the in vitro findings, PRMT1 overexpression in PPE‐treated mice reduced nuclear p65 expression in CDH5^+^ pulmonary cells and decreased phosphorylated p65 levels in whole lung lysates (Figure 9J–L). This was accompanied by reduced γH2AX levels and lower transcript levels of inflammation‐associated markers, including *Icam‐1*, *Ccl‐2*, *Il‐1α*, and *Tnf‐α* (Figure 9M). These findings collectively highlight the protective role of endothelial PRMT1 in COPD by mitigating inflammation and endothelial dysfunction by modulating p65/NF‐κB signaling activation. This underscores the importance of maintaining pulmonary endothelial homeostasis in disease progression.

## 3 Discussion

This study is the first to establish the critical role of PRMT1 in pulmonary endothelial function and COPD pathogenesis. PRMT1 expression was distinctly reduced in pulmonary ECs of COPD patients and in PPE‐induced COPD models, emphasizing its importance in maintaining vascular integrity. The decline in PRMT1 was strongly associated with endothelial dysfunction, a key factor in COPD progression. The loss of PRMT1 in ECs led to pulmonary hemorrhage, compromised barrier function, increased inflammation, and fibrosis, all of which are hallmarks of COPD‐related vascular pathology. These findings underscore the central role of PRMT1 in safeguarding endothelial integrity and preventing vascular dysfunction.

Endothelial dysfunction disrupts barrier function, promoting leukocyte infiltration, inflammation, and vascular instability, which exacerbates COPD.^[^ ^28^ ^]^ PRMT1 ablation amplified these effects, increasing vascular permeability and inflammation. Transcriptome analysis confirmed significant changes in gene expression in PRMT1‐deficient lungs, including upregulation of immune response genes and downregulation of genes linked to developmental morphogenesis. These alterations correlated with increased macrophage infiltration and endothelial dysfunction, further implicating PRMT1 as a regulator of vascular homeostasis.

Single‐cell RNA sequencing revealed a dynamic shift in endothelial subtypes, with an increase in the inflammatory gCap4 population and a decrease in the developmental gCap1 population in PRMT1‐deficient lungs. The gCap4 subset displayed a pronounced upregulation of proinflammatory genes, particularly those associated with interferon and TNF‐α signaling. These pathways are well‐established drivers of COPD pathology.^[^ ^29^ ^]^ The transition from developmental to inflammatory EC phenotypes corresponds with the heightened sensitivity to inflammatory stimuli observed in PPE‐treated PRMT1^+/−^ mice, manifesting as alveolar destruction, macrophage infiltration, and fibrosis. This shift aligns with well‐established findings on EC plasticity,^[^ ^25^, ^30^ ^]^ underscoring its critical role in maintaining pulmonary homeostasis and highlighting its disruption as a fundamental driver of COPD progression.

Endothelial senescence, a recognized hallmark of COPD,^[^ ^31^, ^32^ ^]^ was another significant finding in PRMT1‐deficient lungs. Senescent ECs exhibited increased SA‐β‐Gal activity, impaired proliferation, and elevated cytokine expression, all of which contribute to impaired vascular regeneration and chronic inflammation. In PPE‐treated PRMT1^+/−^ mice, these senescence markers were prominent, and PRMT1 inhibition in TNF‐α‐treated ECs exacerbated these effects. These results suggest that PRMT1 plays a protective role in preventing senescence and maintaining vascular repair, positioning it as a key regulator of endothelial homeostasis.

A major discovery of this study is PRMT1's regulation of NF‐κB signaling, a central inflammatory pathway. PRMT1 ablation resulted in hyperactivation of NF‐κB, driving ECs toward an inflammatory phenotype. Acute inhibition of PRMT1 amplified TNF‐α‐induced endothelial dysfunction and senescence via NF‐κB activation, while inhibition of NF‐κB mitigated these effects. Conversely, PRMT1 overexpression in ECs, achieved through transfection or AAV‐mediated viral transduction in a COPD model, reduced EC dysfunction and suppressed NF‐κB activation, thereby attenuating inflammation and alleviating the COPD phenotype. These findings indicate that PRMT1 suppresses NF‐κB signaling to maintain endothelial homeostasis and prevent inflammatory shifts. This regulation of NF‐κB may underlie the inflammatory transitions, macrophage infiltration, and lung remodeling observed in PRMT1‐deficient mice, providing a mechanistic link between PRMT1 and COPD pathogenesis. However, a limitation of this study is the lack of additional in vivo experiments to further confirm these findings. Future in vivo studies are crucial to further validate and expand on the role of PRMT1 in NF‐κB signaling. In addition, while our study suggests a potential link between EC inflammation and senescence induced by PRMT1 deficiency, likely mediated by NF‐κB signaling, the direct regulation of endothelial senescence by NF‐κB or other signaling pathways remains to be fully understood. Future investigations into the specific role of PRMT1 in endothelial senescence, particularly in relation to NF‐κB signaling, will provide valuable insights into the pathogenesis and progression of COPD.

Our findings also shed light on the role of aCap cells, a lung‐specific endothelial subtype involved in gas exchange.^[^ ^18^ ^]^ PRMT1 deficiency was associated with upregulation of developmental genes in aCap cells, likely reflecting activation of repair programs.^[^ ^33^, ^34^ ^]^ However, the concurrent inflammatory environment suggests a disrupted balance between repair and inflammation, impairing vascular regeneration and contributing to disease progression. This interplay between repair mechanisms and inflammation further emphasizes the importance of PRMT1 in maintaining vascular health. Collectively, our study demonstrates that PRMT1 is essential for the maintenance of endothelial integrity and pulmonary homeostasis. Its role in suppressing NF‐κB‐mediated inflammation, preventing senescence, and regulating endothelial plasticity positions PRMT1 as a critical safeguard against COPD progression. By maintaining quiescent endothelial states and preventing inflammatory transitions, PRMT1 preserves pulmonary vascular function and prevents chronic remodeling.

Despite these advances, further research is required to fully understand the intercellular interactions influenced by PRMT1. While our study identified cell‐intrinsic mechanisms underlying the inflammatory shift in ECs, the role of intercellular communication, particularly between ECs and macrophages, remains unclear. Future investigations into these interactions will provide a more comprehensive understanding of PRMT1's regulatory network. Additionally, exploring PRMT1's systemic effects and its role in other vascular beds may reveal broader therapeutic implications.

In conclusion, PRMT1 emerges as a pivotal regulator of pulmonary endothelial integrity and homeostasis. By suppressing NF‐κB activation and preventing inflammatory shifts, PRMT1 protects against endothelial dysfunction, inflammation, and senescence in COPD. These findings position PRMT1 as a promising therapeutic target for mitigating COPD progression and restoring vascular health.

## 4 Experimental Section

### Mice Experiments

*Prmt1* ^fl/fl^; *Cdh5* ^ERT2–Cre^ mice were generated through the breeding of *Prmt1* ^fl/fl^ mice with *Cdh5* ^ERT2–Cre^ mice kindly provided by Dr. Kyu Young Koh (Center for Vascular Research, Institute for Basic Science). The ablation of PRMT1 in ECs was induced by the intraperitoneal injection of tamoxifen (Sigma, no. T5648‐5G) dissolved in 10% ethanol (Merck, no. 1.00938.1011) and 90% Corn oil (Sigma, no. C8267) at a concentration of 10 µg mL^−1^, and 250 µL of this mixture was administered per mouse, four times for a week. To maintain the KO genotype, mice were fed with tamoxifen‐containing chow (Envigo, no. TD 130859) for the remaining experimental time. The *Prmt1* ^fl/+^ and *Cdh5* ^ERT2‐Cre^ mice were administrated with 50 µL of vehicle served as the control group (Con). To induce COPD, mice were intranasally injected a single dose of 50 µL of PPE (Sigma, no. E7885) dissolved in PBS at a concentration of 1 mg mL^−1^ per animal. Only male mice were used for all experiments in this study. For animal studies, drug administration was approved by the Institutional Animal Care and Use Committee of Sungkyunkwan University School of Medicine and carried out by the ethical guideline (the protocol number: SKKUIACUC 2020‐04‐14‐1).

### Cell Culture, Infection, Transfection, and Immunostaining

Human umbilical vein endothelial cells (HUVECs, American Type Culture Collection (ATCC), no. CRL‐1730) were cultured in F‐12K medium (ATCC, no. 30‐2004) supplemented with 10% fetal bovine serum (FBS), 100 µg mL^−1^ Heparin (Sigma, no. H3393‐100KU), 30 µg mL^−1^ endothelial cell growth supplement (Corning, no. 354006), and antibiotics. HUVECs used in this study were from passages 4–10. The adenoviruses expressing control sh‐scrambled (Con) and sh*PRMT1* (5′‐GCAACTCCATGTTTCACAATC‐3′) were generated as previously described.^[^ ^9^ ^]^ To knock‐down PRMT1, HUVECs were infected with adenovirus for 48 h. Human lung endothelial cells (HULEC‐5a, ATCC, no. CRL‐3244) were cultured in MCDB131 (Gibco, no. 10372019) supplemented with 10% FBS, 10 ng mL^−1^ epidermal growth factor (Gibco, no. PHG0311), 1 µg mL^−1^ Hydrocortisone (Sigma, no. H0888), 10 mm Glutamine (Sigma, no. G7513), and antibiotics. For PRMT1 overexpression, HULECs were transfected with either pcDNA3.1 vector or pcDNA3.1‐HA‐PRMT1 using polyethylenimine (1 mg mL^−1^, Sigma‐Aldrich). Immunostaining of cells was performed as previously described with a minor modification.^[^ ^10^ ^]^ Briefly, HUVECs or HULECs were fixed with 4% paraformaldehyde (PFA) for 15 min, followed by permeabilization with 0.1% Triton X‐100 and blocking with 3% bovine serum albumin (BSA) for 30 min. Primary antibodies and fluor‐conjugated secondary antibodies used in this study are listed in Table S2 in the Supporting Information.

### Senescence Associated β‐Galactosidase Staining

Detection of senescence associated‐β‐galactosidase (SA‐β‐Gal) activity was performed using senescence β‐galactosidase staining kit (Cell Signaling, no. 9860). Briefly, HULECs or lung frozen sections were fixed with fixative solution and incubated with staining solution (pH 6.0) at 37 °C without CO₂ overnight. After that, HULECs or lung sections were washed twice with PBS prior to imaging.

### Nuclear Fractionation

HULECs were incubated on ice for 15 min with Buffer A (40 mm Tris‐HCl pH7.4, 10 nm NaCl, 1 mm ethylenediaminetetraacetic acid (EDTA), 1 mm dithiothreitol DTT). Following this, 10% nonidet P‐40 was added briefly to lyse the cells. After centrifugation, the supernatant was collected for cytoplasmic fraction. The pellet was resuspended in Buffer B (40 mm Tris‐HCl pH7.4, 420 mm NaCl, 10% glycerol, 1 mm EDTA, 1 mm DTT). After centrifugation, the supernatant containing nuclear fraction was collected for further analysis.

### Echocardiography

Echocardiographic analysis was performed using a sonics Vevo 2100 machine with a 40‐MHz probe (visual Sonics). M‐mode tracing was utilized to measure the percentage of FS and EF of the LV. Pulmonary artery flow was assessed using PW Doppler Mode, with measurements of the PAT and PET. Electrocardiogram recordings were obtained from mice under 2% isoflurane anesthesia in the prone position on a warm surface.

### Lung Mechanics Measurement

Lung mechanics were evaluated using a computer‐controlled small‐animal ventilator (FlexiVent, SCIREQ, Montreal, QC, Canada) as previously described.^[^ ^35^, ^36^ ^]^ Mice were anesthetized with avertin (250 mg kg^−1^, intraperitoneal injection) and subsequently administered pancuronium bromide (0.8 mg kg^−1^, intraperitoneal) to inhibit spontaneous respiratory efforts. A tracheotomy was performed and an 8 mm metallic tube was inserted into the trachea to establish a secure airway connection. Mice were mechanically ventilated at a frequency of 150 breaths min^−1^ with a tidal volume of 8.7 mL kg^−1^ and a positive end‐expiratory pressure of 2–3 cm H_2_O. TLC, total respiratory system elastance, tissue elastance, and tissue damping were measured using the FlexiVent system (version 5.3; SCIREQ, Montreal, QC, Canada) to characterize lung mechanical properties.

### Forced Expiratory Volume and Forced Vital Capacity Measurement

The forced expiratory volume in 0.05 s (FEV_0.05_) to FVC ratio or forced expiratory volume in 0.1 s (FEV_0.1_)/FVC ratio was determined using the FlexiVent system, integrated with a negative pressure reservoir. After tracheotomy and mechanical ventilation as described above, the lungs were inflated to a pressure of 30 cm H_2_O within 1 s and maintained at this pressure. Following a 0.2 s hold, the ventilator's pinch valve was closed, and after an additional 0.3 s, the shutter valve connected to the negative pressure reservoir was opened, exposing the lungs to the negative pressure. This negative pressure was sustained for 1.5 s to ensure full expiration. The FEV/FVC ratio was calculated using data acquired and analyzed with the FlexiVent software.

### AAV‐Mediated Prmt1 Overexpression

The AAV2‐QuadYFMP vector which incorporated the *Prmt1* gene and *Cd144* promoter (pAAV‐Cd144>mPrmt1[NM_019830.3]:WPRE3), was purchased from VectorBuilder (China). This construct allows for targeted expression of the *Prmt1* under the control of *Cd144* promoter. C57BL/6 mice (8 weeks old, male) were intravenously injected with 1 × 10^12^gc of AAV2‐QuadYFMP in 100 µL of sterile saline via the tail vein. One week following the viral injection, the mice were intranasally administrated with Elastase to induce COPD for further 2 weeks.

### Tissue Histological and Immunofluorescence Staining

To harvest lung tissues, the right ventricle was perfused with cold PBS followed by fixation with 4% PFA and subsequent paraffin‐embedding or cryoembedding. For the cryoembedding, lung tissues were subjected to cryoprotection through a sucrose‐gradient and embedded in the Tissue‐Tek optimal cutting temperature (Sakura, no. 4583), followed by cryosection. Tissue sections of 7 µm thickness were prepared and subjected to staining with hematoxylin and eosin (H&E, BBC Biochemical), and Masson's trichrome stain (MT, Abcam, no. ab150686), according to the manufacturer's instructions. For immunofluorescence staining, the lung sections were fixed with 4% PFA, permeabilized with 0.5% Triton X‐100, and underwent antigen retrieval using a sodium citrate (pH6.0) solution in boiling water for 10 min. Blocking was performed with a 5% goat serum solution, followed by incubation with the primary and secondary antibodies listed in Table S2 in the Supporting Information.

### Preparation of Single Endothelial Cell Suspension

Eight‐week‐old male mice were utilized to isolate endothelial cells from lung tissue. The lung was dissected without perfusion and a single cell suspension was generated as per the published protocol.^[^ ^37^ ^]^ In brief, the lung was placed in a gentle MACs tube containing 5 mL of digestion buffer, consisting of 0.1% Collagenase type 2 (Worthington Biochemical, no. LS004176), 0.25% Collagenase type 4 (Worthington Biochemical, no. LS004188), and 15 µg mL^−1^ DNase I (Sigma‐Aldrich, no. D4527‐10KU). The tissue was then dissociated using the 37C‐m‐LDK‐1 protocol by the gentleMACSTM Dissociator. Enzyme activity was inhibited by adding 5 mL of washing buffer that contained 0.5% BSA and 1 mm EDTA in PBS. Dissociated cells in suspension were passed through a 70 µm strainer and centrifuged at 300 x *g* for 10 min at 4 °C. Red blood cell lysis was achieved using ammonium‐chloride‐potassium lysing buffer (Gibco, A1049201) for 3 min, and the reaction was stopped with washing buffer. After passing through a 70 µm strainer, the cell suspension was centrifuged again at 300 x *g* for 5 min at 4 °C. The cell suspension was then incubated with a mixture of antibodies containing FITC‐CD45 (1:200, BD Pharmingen, no. 553080), PE‐Ep‐CAM (1:200, BioLegend, no. 118206), APC‐CD31(1:200, BioLegend, no. 102410), fixable viability dye (1:200, eBioscience, no. 65‐2860‐40) for 15 min at 4 °C in the dark condition. After centrifugation, the cell was resuspended in washing buffer and sorted by Fluorescent activated cell sorting.

### Transmission Electron Microscopy

The procedure involved the perfusion of the mouse right ventricle with cold PBS followed by incubation in a fixative solution provided by Match Finder Inc. (South Korea) at 4 °C for overnight. The resulting TEM images were acquired by Match Finder Inc. (South Korea).

### TUNEL Assay

TUNEL assay was performed to investigate the cell death phenotype in lung tissues, according to the manufacturer's protocol (Invitrogen, no. C10246) using the Click‐iT TUNEL Alexa Fluor kit. In brief, paraffin‐embedded lung tissues were sectioned at a thickness of 5 µm and the deparaffinized tissues were incubated with TdT enzyme in a humidified chamber at 37 °C for 1 h. The resulting signals were developed with Click‐iT reaction buffer for 30 min at RT while protected from light. Following washing with 3% BSA solution, nuclei were counterstained with DAPI.

### Protein Extraction and Analysis

For protein sampling, homogenized mouse lungs, HUVECs, and HULECs were lysed utilizing radioimmunoprecipitation assay buffer (Intron, no. IBS‐BR002) buffer and subsequently subjected to standard western blotting analysis. The protein levels were quantified using signal density analysis by using the National Institutes of Health Image J tool and normalized to the loading control. Antibodies used in immunoblotting are listed in Table S2 in the Supporting Information.

### Immunoprecipitation

Immunoprecipitation analyses were performed following established procedures.^[^ ^38^ ^]^ In brief, cellular lysis was carried out using an extraction buffer (10 nm Tris‐HCl (pH 8.0), 150 mm NaCl, 1 mm EDTA, 1% Triton X‐100, 10 mm NaF, 1 mm sodium vanadate, and 0.1 mm phenylmethylsulfonyl fluoride), supplemented with a complete inhibitor cocktail. The resultant precleared cell extracts were subjected to an overnight incubation at 4 °C with specific antibodies. Subsequently, immunocomplexes were precipitated using Dynabeads (Invitrogen, no. 10003D) in accordance with the manufacturer's recommended protocol.

### RNA Extraction and Quantitative PCR Analysis

RNA analysis was performed as previously described with minor modifications.^[^ ^12^ ^]^ Total RNA was extracted from HUVECs, HULECs, and mouse tissues using TRIzol (Invitrogen, no. 15596‐018) in accordance with the manufacturer's guidelines. Complementary DNA (cDNA) samples were synthesized with the PrimeScript RT reagent kit (TaKaRa, no. RR037A), following the manufacturer's protocol. Quantitative analysis of total mRNA expression levels was performed using TaqMan Green Premix Ex Taq (TaKaRa, no. RR420A) on a Thermal Cycler Dice Real Time System machine (TaKaRa, no. TP800), according to the manufacturer's instructions.

### RNA Sequencing Analysis

A previously published RNA sequencing dataset, deposited in GEO under the accession GSE57148, was used to assess the expression of Prmt1 and other genes in the lung samples from normal and COPD patients. For whole lung RNA sequencing, 8‐week‐old mice with control (Con) or endothelial‐specific Prmt1 KO were treated with either vehicle or tamoxifen for 1 week, with tamoxifen administered four times per week. Following a 2‐week waiting period, lungs were harvested after perfusion with cold PBS. Total mRNA was extracted using easy‐BLUE reagent according to the manufacturer's instructions. High‐throughput sequencing was performed using Illumina NEstWEq500 (Ebiogen, Korea) as single‐end 75 sequencing. The analysis for RNA sequencing data was performed by using ExDEGA v1.61 (e‐Biogen) and ClueGO/CluePedia v2.5.10,^[^ ^39^ ^]^ EnrichmentMap v3.3.5,^[^ ^40^ ^]^ and GeneMania v3.5.2^[^ ^41^ ^]^ plugin from Cytoscape v3.9.1^[^ ^42^ ^]^ software. Differentially expressed genes (DEGs) were identified based on the criteria of fold change > 1.3, normalized data(log2) > 2, and *p*‐value < 0.05, and were assessed for biological processes using Gene Set Enrichment Analysis with the MSigDB database v6.1.

### Single Cell RNA Sequencing Data Processing and Clustering

Previously published scRNA‐seq data deposited in GEO under accessions GSE168299 and GSE173896 are used to further assess the expression of PRMT1 in ECs. All single cell‐RNA‐seq analyses were performed in an R 4.2.2 environment using Seurat v4.3.1.^[^ ^43^ ^]^ Prior to processing, reads with mitochondrial genes comprising less than 5% of the total were filtered out. To eliminate low‐quality cells, only those expressing between 500 and 3500 features, and genes expressed in at least three cells, were retained for further analysis. To exclude doublets, samples with scores less than 0.434 were selected using the scDblFinder v1.13.2 pipeline.^[^ ^44^ ^]^ The following steps were performed using Seurat, data was log‐normalized and scaled with exclusion of unique molecular identifiers (UMIs). A set of 2000 highly variable genes from all samples was utilized to integrate the control (Con) and KO samples into a single Seurat object using the IntegrateData function. Subsequently, principal component analysis reduction was performed with 30 npcs, and ten components were used for embedding UMAP clusters with a resolution 0.5. Cell types were assigned to cell clusters by evaluating gene expressions within each cluster using the FindAllMarkers function and subsequent manual annotation. Pathway analysis of samples was performed using singleseqgset v0.1.2.9, with hallmark gene set collection from broad institute GSEA.^[^ ^45^ ^]^ For figure visualization such as dop plots, violin plots and heatmap, the Seurat object was converted into Scanpy v1.9.7 AnnData in Python 3.10.0.^[^ ^46^ ^]^

### Cell to Cell Communication Analysis

Cell to cell communication was performed using CellChat v1.5.0^[^ ^47^ ^]^ pipeline in an R 4.2.2 using seurat based clustering and corrected normalized matrix as inputs. Next, communication probabilities were computed for the “Secreted Signaling”, “Cell‐Cell Contact”, and “ECM Signaling” categories from the CellChat Database and communications with fewer than ten occurrences were excluded. For the main figure, manual curation of the results was performed using biological knowledge.

### Trajectory Analysis

These steps were performed using scFates v1.0.6,^[^ ^48^ ^]^ a python package built in continuity of the R package. Trajectory inference was performed on general capillary cells only and the Seurat object was converted into Scanpy AnnData in Python 3.10 for scFates analysis. Palantir pipeline was used to obtain a multiscale diffusion space and generate embeddings. The process includes plotting results, learning trees with SimplePPT, selecting roots, computing pseudotime, and representing trajectories and trees on embeddings and dendrograms. A root was manually selected on the merged tree, followed by the computation of pseudotime using scFates (tl.pseudotime, default parameters). Subsequently, the dendrogram was generated based on the pseudotime values using scFates (tl.dendrogram).The method also covers tests and fits for features associated with the tree, plotting single features and differential expression analysis, and analyzing bifurcation and fate‐specific modules.

### Gene Ontology Enrichment Analysis of Differentially Expressed Genes

The cytoscape v3.9.1^[^ ^42^ ^]^ cluego^[^ ^39^ ^]^ plugin was used to visualize enriched pathways associated with biological pathway database. In brief, biological GO terms were explored with medium specificity and a kappa score of 0.4. An enrichment/depletion method with a two‐sided hypergeometric test was applied with Bonferroni step down for each *p*‐value calculation. Enriched pathways with a *p*‐value < 0.05 were considered significant. GSEA was performed to extract knowledge of overrepresented gene ontology terms for various functional processes and signaling pathway between each sample. Visualization of significantly enriched GO terms of functional process and signaling pathway between samples was done with the cytoscape plugin, EnrichmentMap.^[^ ^40^ ^]^ The mapping of gene expression levels was done using the GeneMania plugin.^[^ ^41^ ^]^ All GO terms of network in the analysis were filtered with *p*‐value below 0.05 based on the pathway score.

### Imaging

Confocal images were taken by LSM‐710 system (Carl Zeiss) or BioTek Cytation 10. Microscopy photographs were taken by Nikon ECLIPS TE‐2000U microscope. Imaging analysis was conducted by ZEN or ImageJ software.

### Statistical Analysis

Here, the data are expressed as means ± SD, or 95% confidence interval as indicated. The values were subjected to unpaired two‐tailed Student's *t*‐test for statistical analysis. For multiple comparisons, analysis of variance (one way ANOVA) test was employed, followed by Dunnett's testing, utilizing the GraphPad Prism software, version 8.0.2. A *p*‐value of less than 0.05 was considered statistically significant.

## Conflict of Interest

The authors declare no conflict of interest.

## Author Contributions

T.T.V.T. and Y.J. contributed equally to this work. T.T.V.T., Y.J., S.K., G.‐U.B., and J.‐S.K. conceived and designed the project. T.T.V.T., Y.J., S.K., and J.E.Y. performed the experiments. Y.J. carried out bioinformatics analysis. T.T.V.T., Y.J., J.L., J.E.Y., W.L., G.‐U.B., and J.‐S.K. analyzed the data and wrote the paper.

## Supporting information



Supporting Information

## Data Availability

The data that support the findings of this study are available from the corresponding author upon reasonable request.
