# Supplementary material for: PRMT1 Ablation in Endothelial Cells Causes Endothelial Dysfunction and Aggravates COPD Attributable to Dysregulated NF‐κB Signaling
Source: Adv Sci (Weinh). 2025 Mar 26;12(19):2411514. doi: 10.1002/advs.202411514 (PMC12097043; doi:10.1002/advs.202411514)
Supplement: Supplementary file 1 — Supporting Information [file ADVS-12-2411514-s001.docx]

Supporting Information

PRMT1 Ablation in Endothelial Cells Causes Endothelial Dysfunction and Aggravates COPD Attributable to Dysregulated NF-κB Signaling

Thi Thuy Vy Tran, Yideul Jeong, Suwoo Kim, Ji Eun Yeom, Jinwoo Lee, Wonhwa Lee, Gyu-Un Bae^*^, Jong-Sun Kang^*^


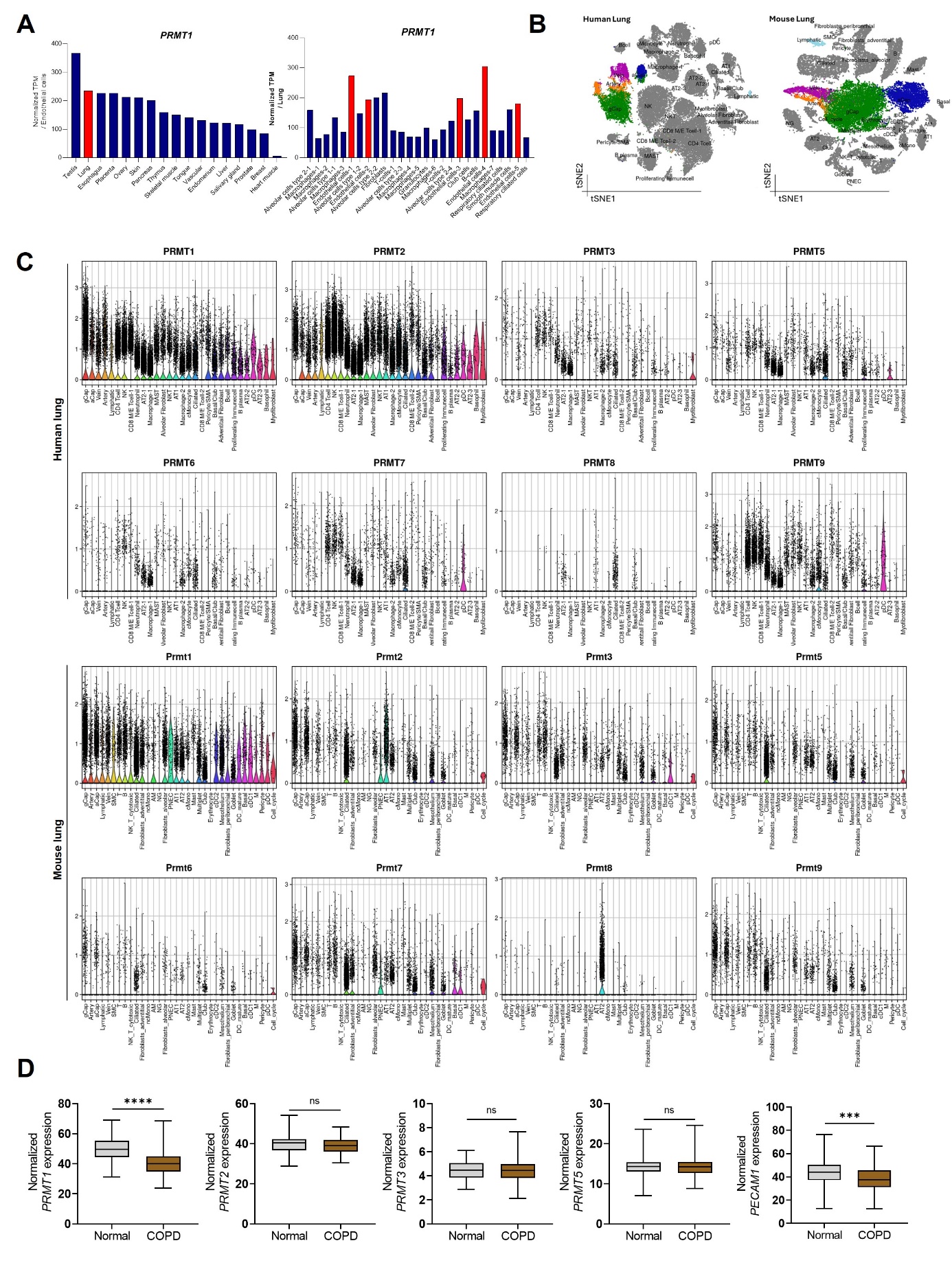


**Figure S1.** Expression of *PRMT*s in human and mouse lung datasets. A) Expression pattern of *PRMT1* in various human tissues and in distinct human pulmonary cell types. B) tSNE projections of pulmonary human cells (left, GSE173896) and mouse cells (right, GSE168299). C) Expression of *PRMT*s in various cell types in lung tissues from human (upper) and mouse (lower). D) Normalized genetic expression of *PRMT1*, *PRMT3*, *PRMT5*, and *PECAM1* in lung tissue of healthy individuals (n=91) and COPD patients (n=98) (GSE57148).


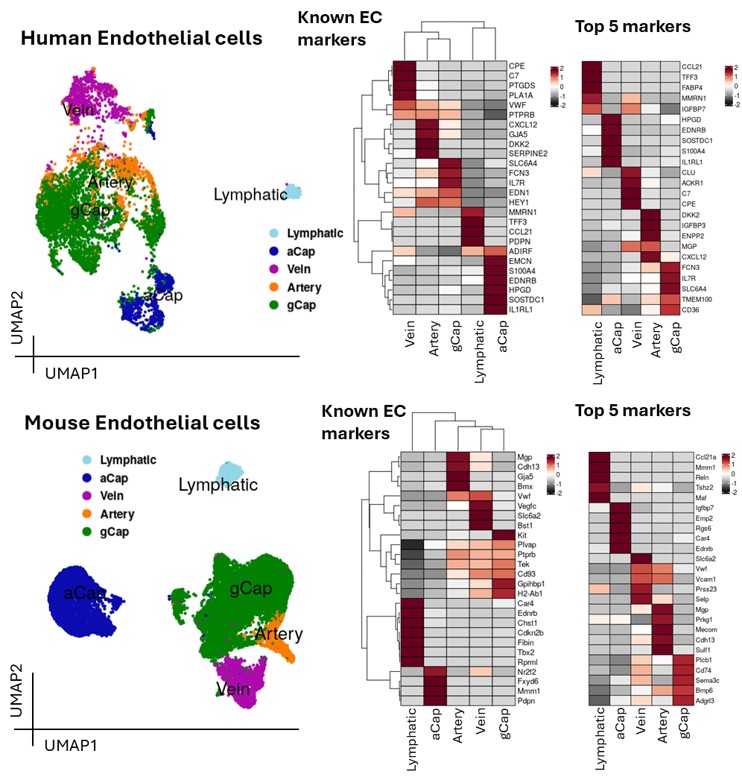


**Figure S2.** UMAP analysis of EC subtypes in human mouse lung datasets. UMAP plots of human (top, GSE173896) and mouse (bottom, GSE168299) ECs, color-coded by subtypes (Lymphatic, aCap, Vein, Artery, and gCap). The heatmaps illustrate the expression of established EC markers (left) and the top five subtype-specific markers (right) for both human and mouse. Color intensity indicates relative gene expression levels.


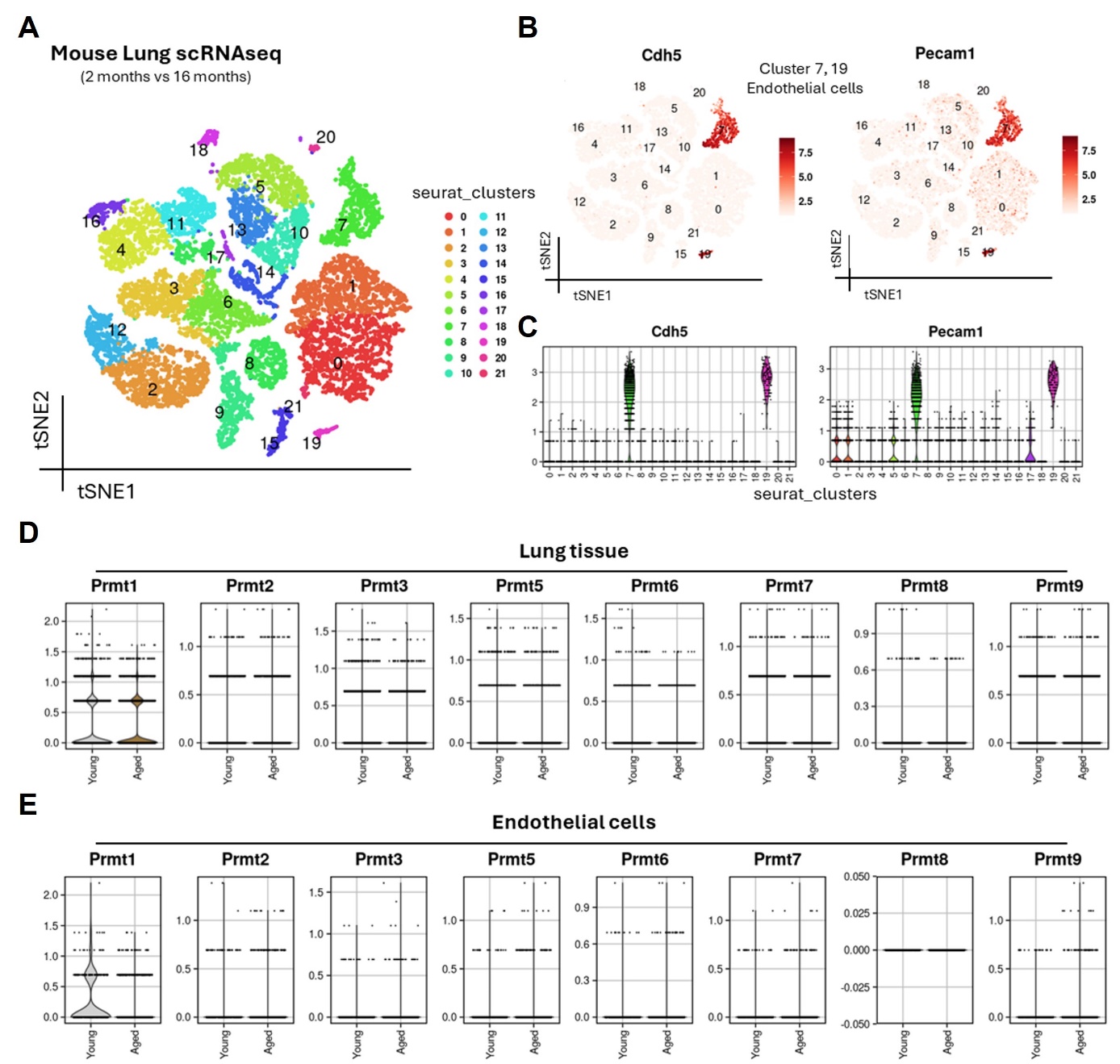


**Figure S3.** t-SNE analysis and *Prmt*s expressions in pulmonary cells from murine young and aged datasets. A) t-SNE projections of 21 cell clusters derived from young and aged lung tissues. B) t-SNE projection showing the identification of cluster 7 and 19 as ECs based on elevated *Cdh5* and *Pecam1* expression. C) Violin blot of *Cdh5* and *Pecam1* expressions across 21 cell clusters. D, E) Box plots for comparing *Prmt*s expression in total lung tissue (D) and specifically within ECs (E) between young and aged mice.


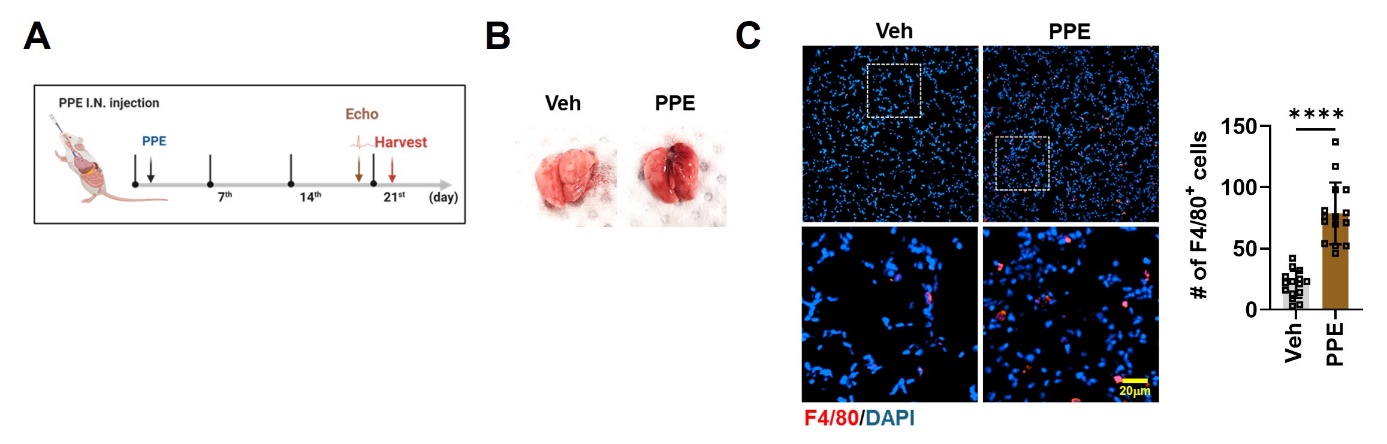


**Figure S4.** Generation of COPD mouse model and macropahge filtration in pulmonary alveoli. A) Scheme of PPE injection for generating PPE-induced COPD mouse models. B) Photographs of isolated lungs from vehicle (Veh) or PPE-treated (PPE) mice. C) Confocal images of pulmonary alveolar stained for macrophage marker F4/80 (red) and DAPI (blue). Scale bar=20µm. Quantification of F4/80^+^ cells in the alveolar of veh or PPE mice. Data are means ±SD. Student’s t-test. ****p<0.0001.


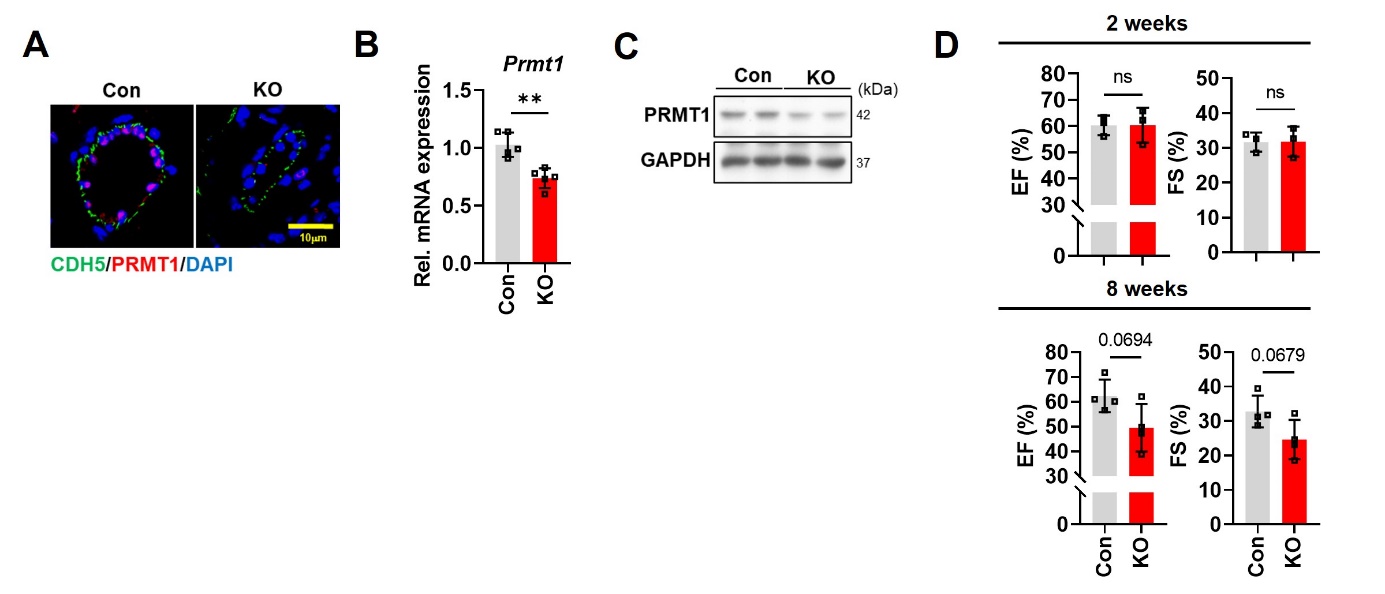


**Figure S5.** Endothelial Prmt1 expression and cardiac function assessment in Con and KO mice. A) Representative confocal images of pulmonary blood vessel, stained for EC marker (CDH5, green), PRMT1 (red) and counterstained with DAPI (blue). Scale bar=10µm. B) qRT-PCR analysis of *Prmt1* expression using whole-lung mRNA of Con or KO mice (n=5). C) Immunoblot analysis for protein expression level of PRMT1 in the lung of Con or KO mice (n=2). D) Electrocardiographic assessment of ejection fraction (EF) and fraction shortening (FS) in Con, 2-week KO (n=3) or 8-week KO mice (n=4). Data for all panels are means ±SD. Student’s t-test. ns=not significant, **p<0.01.


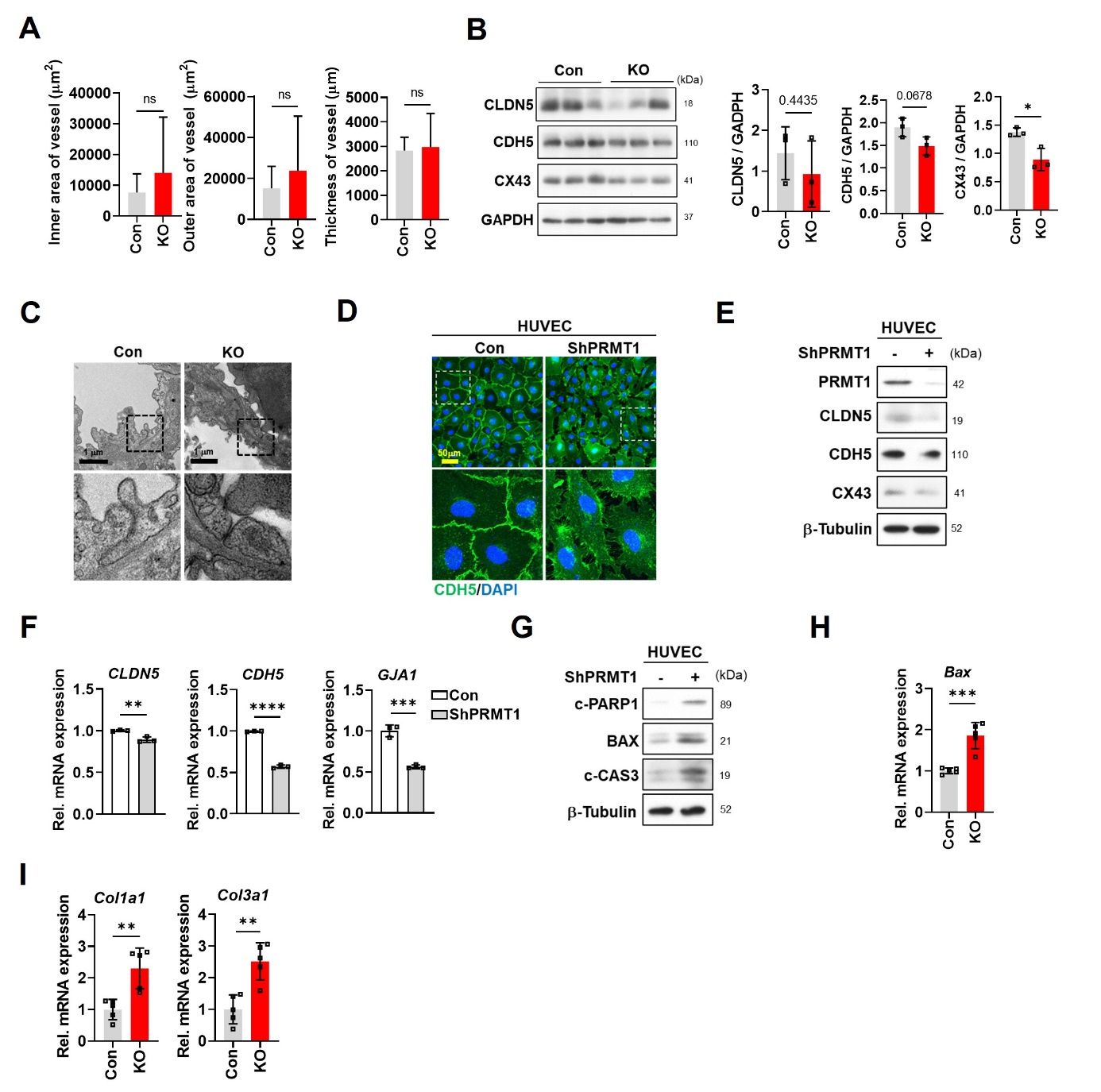


**Figure S6.** Evaluation of endothelial junction and cell death in PRMT1 deficiency in mouse and HUVECs. A) Measurement of inner area, outer area, and thickness of vessels in Con or 2-week KO mice. B) Immunoblot analysis for CLDN5, CDH5, CX43, and GAPDH protein levels using lung lysates from Con or KO mice (n=3). Quantification of relative protein level of CLDN5, CDH5, and CX43 to GAPDH. C) TEM images displaying pulmonary vascular endothelial junctions in Con or KO mice. D) Representative confocal images of adenovirus construct (Con) or adenovirus carrying sh*PRMT1*-infected HUVECs (shPRMT1) for 48 hours, immuno-stained for endothelial junction (CDH5, green) and counterstained with DAPI (blue). Scale bar=50µm. E) Immunoblot analysis of protein expression levels of PRMT1, CLDN5, CDH5, CX43 and β-Tubulin of Control or shPRMT1 HUVECs. F) qRT-PCR analysis of endothelial junction markers, including *CLDN5*, *CDH5*, or *GJA1* expression of Con or shPRMT1 HUVECs. G) Immunoblot analysis of protein expression levels of cleaved PARP1 (c-PARP1), BAX, cleaved Caspase 3(c-CAS3) and β-Tubulin of Control or shPRMT1 HUVECs. H, I) qRT-PCR analysis of cell death marker (*Bax*) and fibrosis markers (*Col1a1*, *Col3a1*) in Con and KO mice (n=5). Data for all panels are means ±SD. Student’s t-test. ns=not significant, *p<0.05, **p<0.01, ***p<0.001, ****p<0.0001.


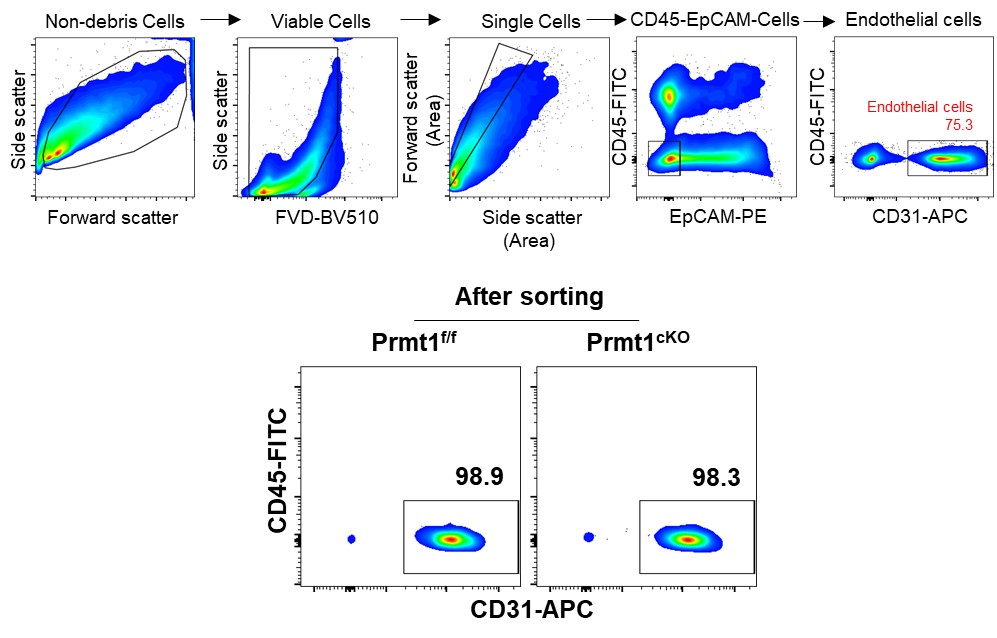


**Figure S7.** Flow cytometric analysis of pulmonary ECs isolation. Debris and doublets were removed, and then ECs were assessed as lineage-negative (CD45 negative/EpCAM negative) CD31-positive cells. Gates were determined on the basis of Fluorescence Minus One controls (FMOs). FVD: Fixable Viability Dye.


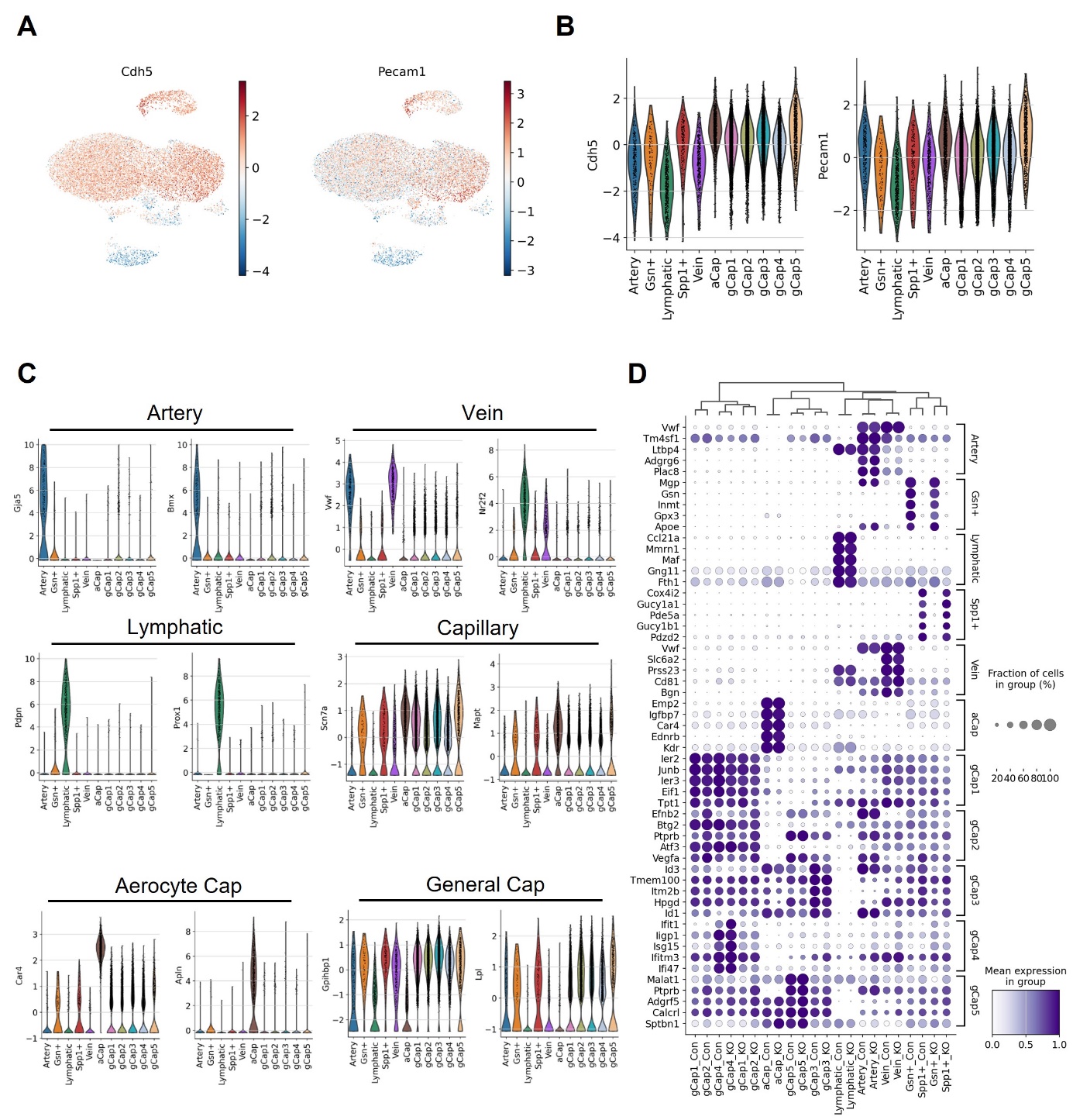


**Figure S8.** Gene expression of endothelial markers in pulmonary EC subtypes. A, B) Expression of endothelial marker genes (*Cdh5*, *Pecam1*) in pre-filtered pulmonary ECs visualized as feature plots (A) and violin plots (B). C) Visualization of specific markers for EC sub-populations as arterial (*Gja5*, *Bmx*), venous (*Vwf*, *Nr2f2*), lymphatic (*Pdpn*, *Prox1*), as well as general (*Gpihbp1*, *Lpl*) and aerocyte (*Car4*, *Apln*). D) Comparative expression of highly variable genes in Con and KO EC sub-populations.


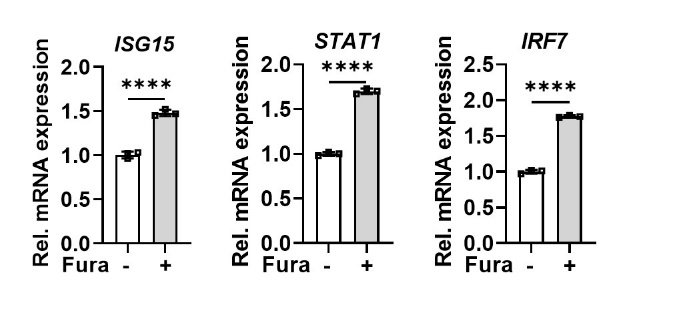


**Figure S9.** Gene expression of inflammatory markers in HUVECs. qRT-PCR analysis of *ISG-15*, *STAT1*, and *IRF-7* mRNA expression levels of HUVECs treated with DMSO or furamidine (Fura, 20µM) for 2 hours. Data are means ±SD. Student’s t-test. ****p<0.0001.


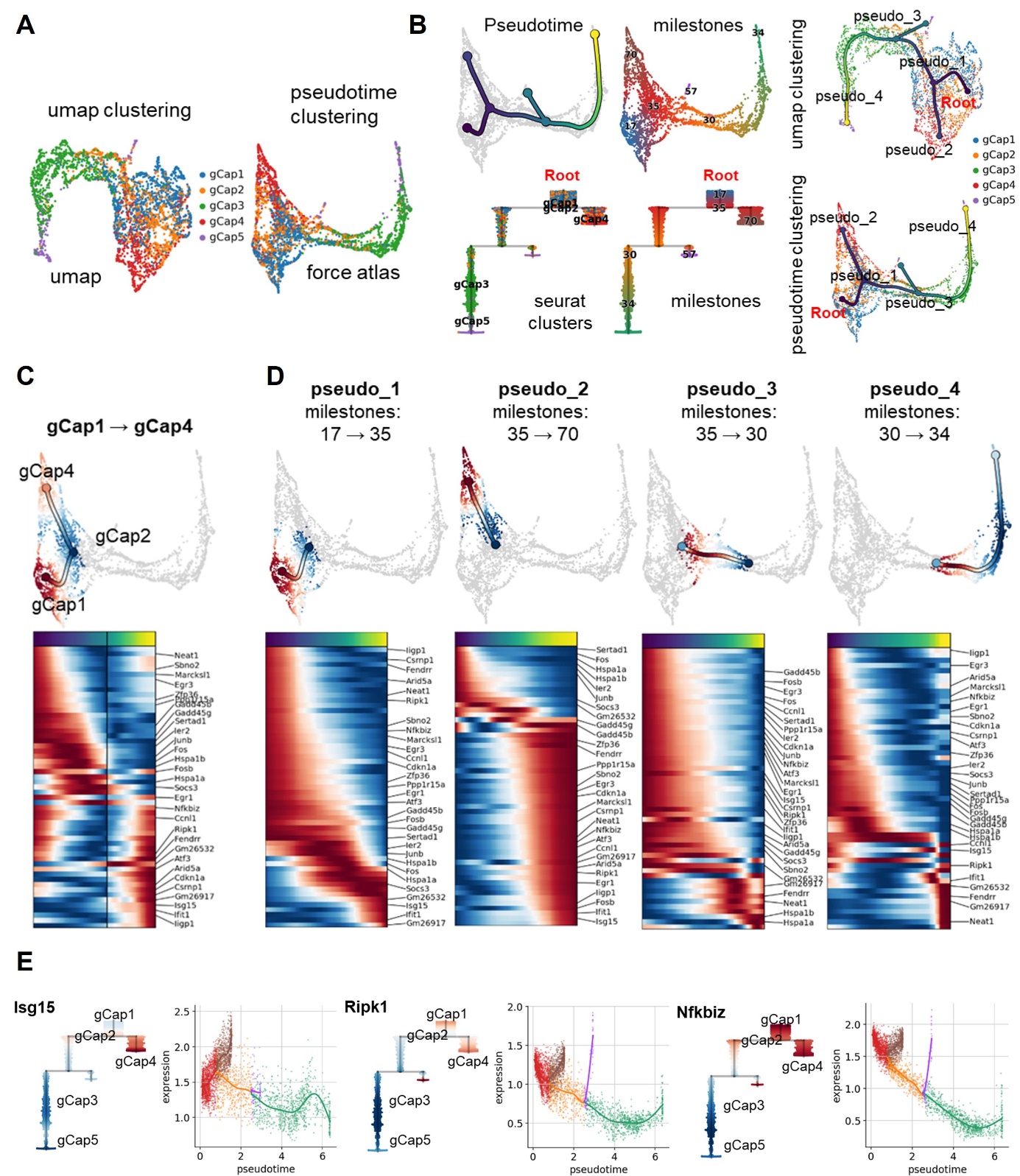


**Figure S10.** Trajectory analysis of pulmonary gCap subtypes. A) Dotplot of pulmonary ECs by UMAP and Force Atlas2(FA) based layouts showing the distinct clustering between gCap sub-clusters. B) Fate mapping and dendrograms recapitulate the branched trajectory of gCap-clusters based on the transcriptional similarity of pseudotime-ordered cells. UMAP and FA plots show the summarization of pseudotimes initiated from the root point. C) Visualization of gene expression patterns for phenotypic changes during gCap1 to gCap4. gCap1 was selected as the initial point. D) Visualization of gene expression patterns for phenotypic changes in batch_1 (milestones 17 to 35), batch_2 (milestones 35 to 70), batch_3 (milestones 35 to 30) and batch_4 (milestones 30 to 34) with 56 variable genes obtained from computational calculations under scFates. The top 25 genes in each batch were shown. E) Dendrograms and gene-expression trends of top ranked genes in gCap4 over pseudotimes.


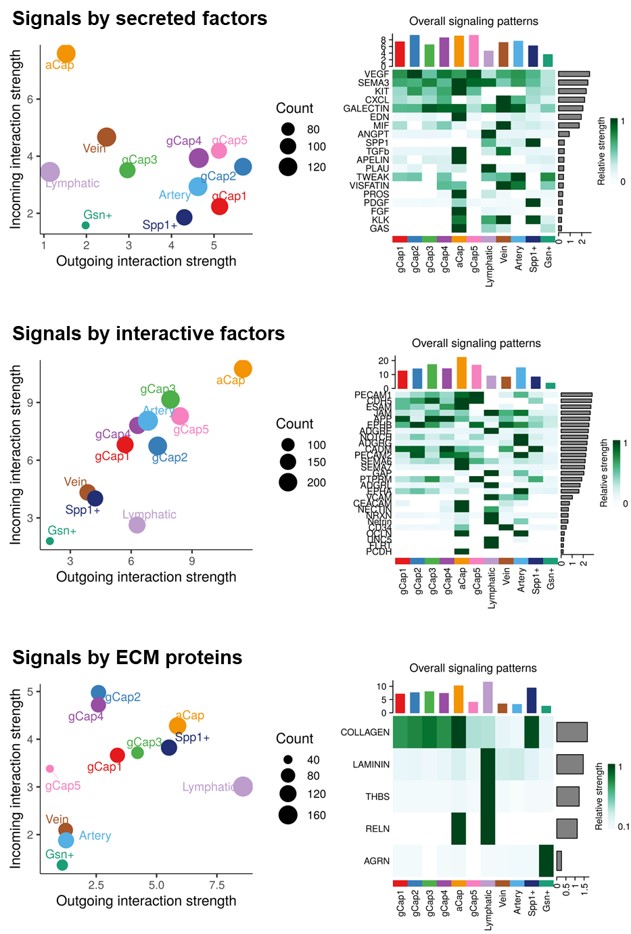


**Figure S11.** Ligand-receptor interaction analysis of pulmonary ECs. PCA plots and heatmaps show the activities for ‘secreted factors,’ ‘interactive factors,’ and ‘ECM factors’ in pulmonary ECs. Dot plots demonstrate the interaction strength for incoming/outgoing factors and heatmaps show the relevant expression of involved genes in each group.


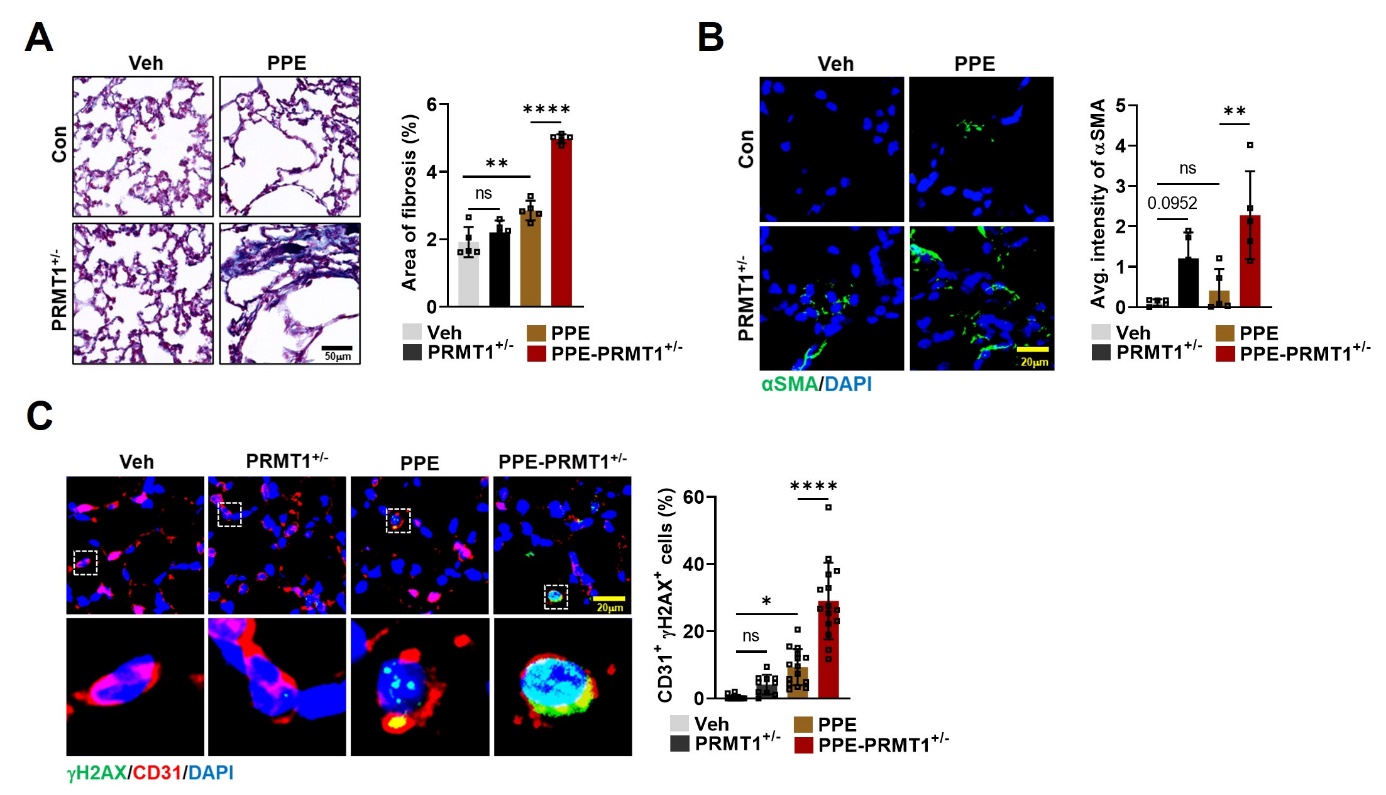


**Figure S12.** Histological analysis of fibrosis and DNA damage in murine lungs. A) Histological analysis of alveolar area using Masson’s Trichrome staining. Scale bar=50µm. Quantification of percentage of fibrosis area in the lung of Veh, PRMT1^+/-^, PPE, and PPE-PRMT1^+/-^mice. B) Confocal images of lung tissue, stained for αSMA (green) and DAPI (blue). Scale bar=20µm. Quantification of mean intensity of αSMA in the lung of Veh, PRMT1^+/-^, PPE, and PPE-PRMT1^+/-^mice. C) Confocal images of lung tissues, stained for DNA damage (γH2AX, green), ECs (CD31, red), and counterstained with DAPI (blue). Scale bar=20µm. Quantification of γH2AX-positive CD31-positive cells in the alveolar areas. Data for all panels are means ±SD. One-way ANOVA. ns=not significant, *p<0.05, **p<0.01, ****p<0.0001.


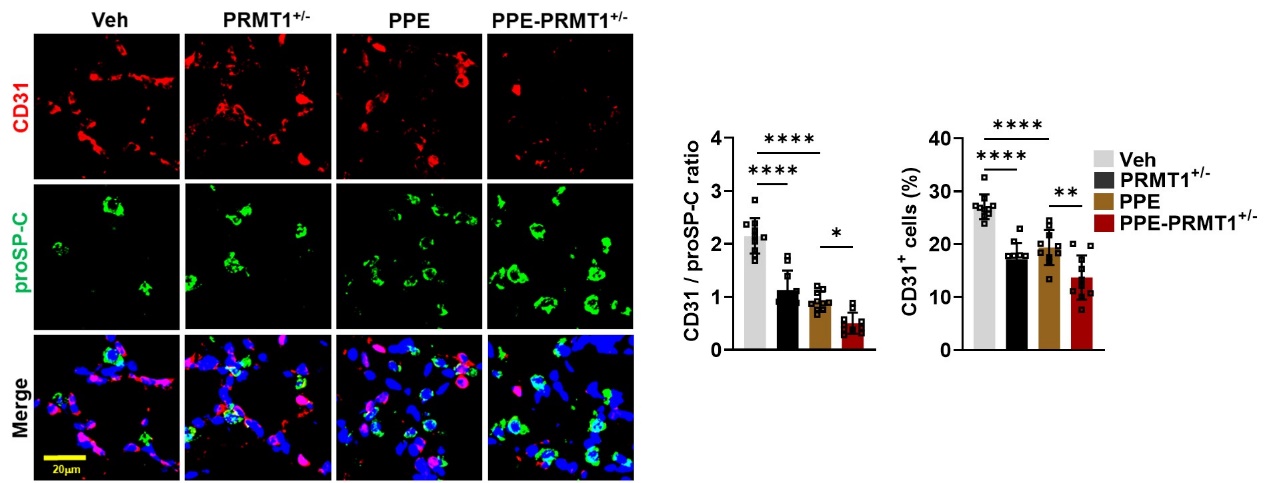


**Figure S13.** Endothelial and alveolar cells staining in lung tissue. Confocal images of alveolar area, stained for ECs (CD31, red), alveolar cell (proSP-C, green), and counterstained with DAPI (blue). Scale bar=20µm. Quantification of CD31-posotive cells in the alveolar and ratio of CD31-positive cells to proSP-C-positive cells in the pulmonary alveolar area of Veh, PRMT1^+/-^, PPE, and PPE-PRMT1^+/-^mice. Data are means ±SD. One-way ANOVA.*p<0.05, **p<0.01, ****p<0.0001.


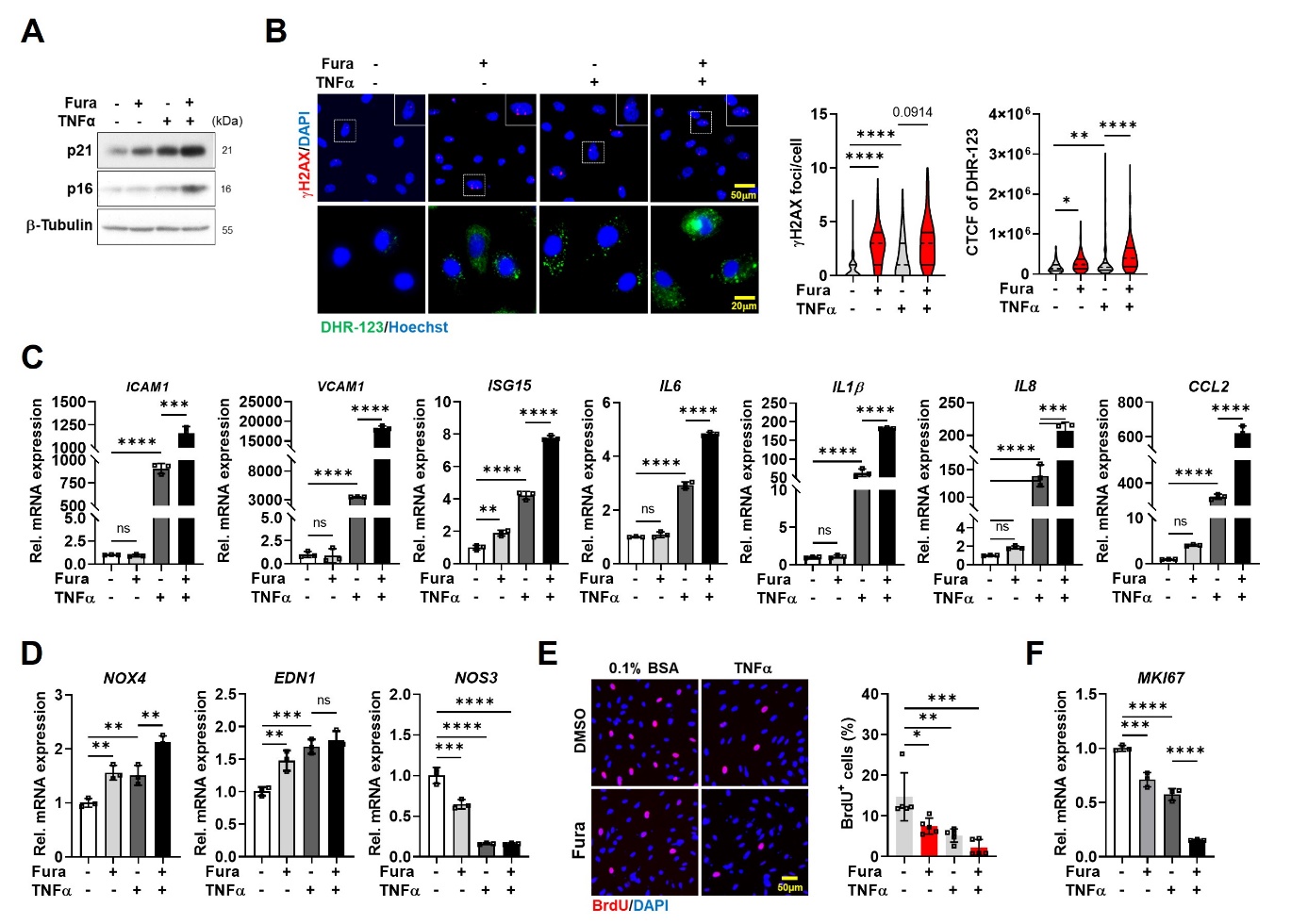


**Figure S14.** In vitro analysis of cellular stress and senescence. A) Immunoblot analysis of p21, p16, and β-Tubulin protein levels of HULECs treated with DMSO, Fura (20μM), TNF-α (50ng/mL), or a combination of Fura and TNF-α for 24 hours. B) Confocal images of γH2AX (upper panel, red), DAPI (blue) and DHR-123(green), Hoechst (blue) (lower panel) in HUVECs treated with DMSO, Fura (20µM), TNF-α (50ng/mL), or a combination of Fura and TNF-α for 24 hours. Scale bar=50µm (upper panel), scale bar=20µm (lower panel). Quantification of the average of the γH2AX foci per cell and the corrected total cell fluorescence (CTCF) of DHR-123. Data represents mean change from baseline (±95% CI). One-way ANOVA. *p<0.05, **p<0.01, ****p<0.0001. C, D) qRT-PCR analysis of *ICAM-1*, *VCAM-1*, *ISG-15*, *IL-6*, *IL-1α*, *IL-8*, *CCL-2*, *NOX4*, *EDN1*, and *NOS3* mRNA expression in HUVECs treated with the same conditions in panel (B). E) Fluorescence microscope images showing BrdU (red) positive HUVECs, and counterstained with DAPI (blue). Scale bar=50µm. Quantification of the percentage of BrdU-positive HUVECs. F) qRT-PCR analysis of *MKI67* mRNA expression in HUVECs. Data for panels from (C) to (F) are means ±SD. One-way ANOVA. ns=not significant, *p<0.05, **p<0.01, ***p<0.001, ****p<0.0001.


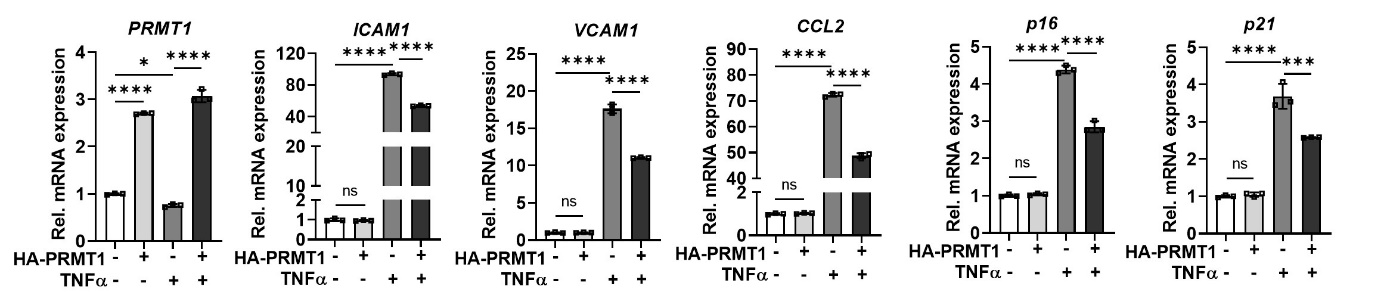


**Figure S15.** Evaluation of *PRMT1*, inflammation, and senescence markers in HULECs. qRT-PCR analysis of *PRMT1*, *ICAM-1*, *VCAM-1*, *CCL-2*, *p16*, and *p21* mRNA level of HULECs treated with pcDNA3.1 vector (Control) or pcDNA3.1-HA-PRMT1 (HA-PRMT1) for 24 hours, followed by treatment of 0.1% BSA or TNF-α (50ng/mL) for 24 hours. Data are means ±SD. One-way ANOVA. ns=not significant, *p<0.05, ***p<0.001, ****p<0.0001.


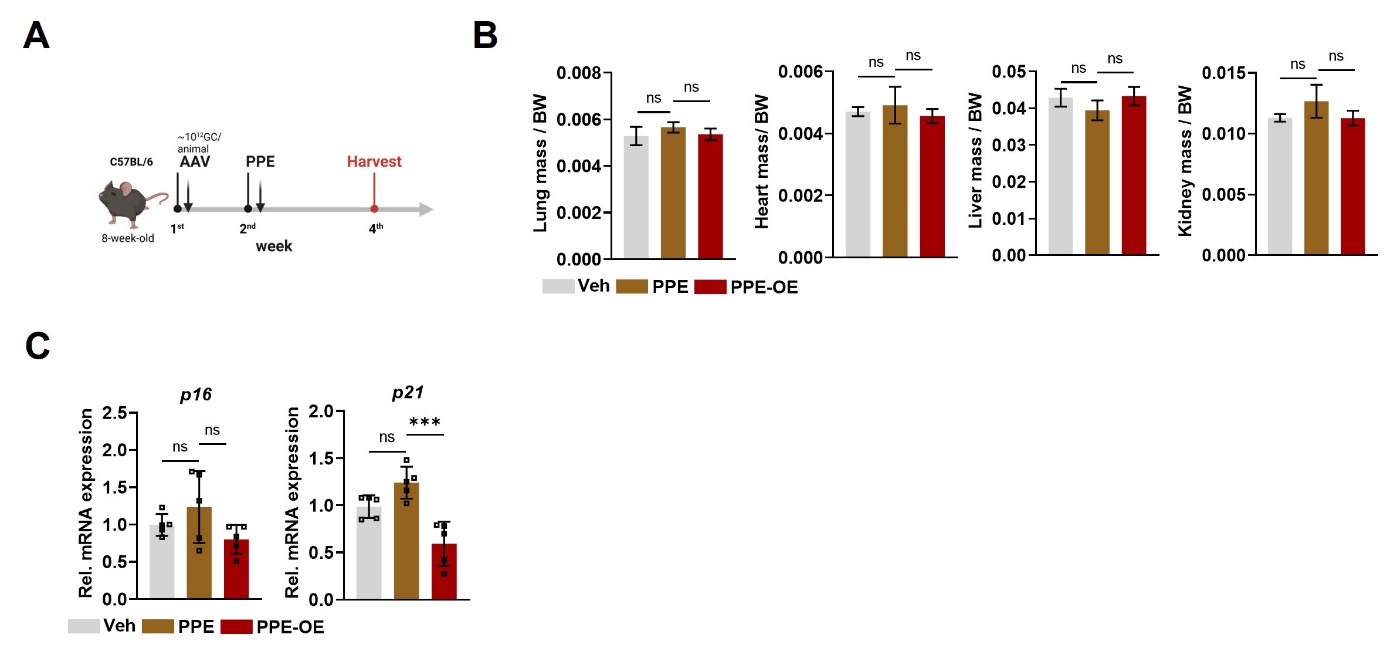


**Figure S16.** Effects of endothelial PRMT1 overexpression on lung and tissue mass in PPE-induced COPD mice. A) Scheme of AAV vector or AAV carrying *Prmt1* gene administration to induce endothelial PRMT1 overexpression, followed by PPE injection to induce COPD. B) Evaluation of the relative lung and other tissues, including heart, liver, and kidney mass to body weight ratio in Veh (n=3), PPE (n=5), and PPE-OE (n=5) mice. C) qRT-PCR analysis of *p16*, and *p21* expression level in the lung from Veh, PPE, or PPE-OE mice (n=5). Data for all panels are means ±SD. One-way ANOVA. ns=not significant, *p<0.05, ***p<0.001.

**Table S1. Primer sequences used in qRT-PCR.**

| Gene symbol |  | Sequence | |
| --- | --- | --- | --- |
| h-*ICAM-1* | Forward | | 5'-TGT-GAC-CAG-CCC-AAG-TTG-TT-3' |
|  | Reverse | | 5'-AGT-CCA-GTA-CAC-GGT-GAG-GA-3' |
| h-*VCAM-1* | Forward | | 5'-CCA-GTT-GAA-GGA-TGC-GGG-AG -3' |
|  | Reverse | | 5'-ATG-ACC-CCT-TCA-TGT-TGG-CT-3' |
| h-*CCL-2* | Forward | | 5'-GTC-TCT-GCC-GCC-CTT-CTG-T-3' |
|  | Reverse | | 5'-TTG-CAT-CTG-GCT-GAG-CGA-G-3' |
| h-*IL-8* | Forward | | 5’-AAG-AAA-CCA-CCG-GAA-GGA-AC -3’ |
|  | Reverse | | 5’-ACT-CCT-TGG-CAA-AAC-TGC-AC-3’ |
| h-*IL-1β* | Forward | | 5'-CCA-CAG-ACC-TTC-CAG-GAG-AAT-G-3' |
|  | Reverse | | 5'-GTG-CAG-TTC-AGT-GAT-CGT-ACA-GG-3' |
| h-*IL-6* | Forward | | 5'-AGA-CAG-CCA-CTC-ACC-TCT-TCA-G -3' |
|  | Reverse | | 5'-TTC-TGC-CAG-TGC-CTC-TTT-GCT-G-3' |
| h-*ISG-15* | Forward | | 5'-CTC-TGA-GCA-TCC-TGG-TGA-GGA-A-3' |
|  | Reverse | | 5'-AAG-GTC-AGC-CAG-AAC-AGG-TCG-T-3' |
| h-*NOX4* | Forward | | 5'-GCA-GGA-GAA-CCA-GGA-GAT-TG-3' |
|  | Reverse | | 5'-CAC-TGA-GAA-GTT-GAG-GGC-ATT-3' |
| h-*NOS3* | Forward | | 5‘-ACC-CTC-ACC-GCT-ACA-ACA-T-3’ |
|  | Reverse | | 5‘-GCC-TTC-TGC-TCA-TTC-TCC-A-3’ |
| h-*EDN1* | Forward | | 5‘-CTA-CTT-CTG-CCA-CCT-GGA-CAT-C-3’ |
|  | Reverse | | 5‘-TCA-CGG-TCT-GTT-GCC-TTT-GTG-G-3’ |
| h-*MKI67* | Forward | | 5’-GAA-AGA-GTG-GCA-ACC-TGC-CTT-C-3’ |
|  | Reverse | | 5’-GCA-CCA-AGT-TTT-ACT-ACA-TCT-GCC-3’ |
| h-*STAT1* | Forward | | 5’-ATG-GCA-GTC-TGG-CGG-CTG-AAT-T-3’ |
|  | Reverse | | 5’-CCA-AAC-CAG-GCT-GGC-ACA-ATT-G-3’ |
| h-*IRF-7* | Forward | | 5’-CCA-CGC-TAT-ACC-ATC-TAC-CTG-G-3’ |
|  | Reverse | | 5’-GCT-GCT-ATC-CAG-GGA-AGA-CAC-A-3’ |
| h-*CLDN5* | Forward | | 5’-ATG-TGG-CAG-GTG-ACC-GCC-TTC-3’ |
|  | Reverse | | 5’-CGA-GTC-GTA-CAC-TTT-GCA-CTG-C-3’ |
| h-*CDH5* | Forward | | 5’-GAA-GCC-TCT-GAT-TGG-CAC-AGT-G-3’ |
|  | Reverse | | 5’-TTT-TGT-GAC-TCG-GAA-GAA-CTG-GC-3’ |
| h-*GJA1* | Forward | | 5’-GGA-GAT-GAG-CAG-TCT-GCC-TTT-C-3’ |
|  | Reverse | | 5’-TGA-GCC-AGG-TAC-AAG-AGT-GTG-G-3’ |
| h-*p16* | Forward | | 5’-CTC-GTG-CTG-ATG-CTA-CTG-AGG-A-3’ |
|  | Reverse | | 5’-GGT-CGG-CGC-AGT-TGG-GCT-CC-3’ |
| h-*p21* | Forward | | 5’-AGG-TGG-ACC-TGG-AGA-CTC-TCA-G-3’ |
|  | Reverse | | 5’-TCC-TCT-TGG-AGA-AGA-TCA-GCC-G-3’ |
| m-*Prmt1* | Forward | | 5’-ACC-CTC-ACA-TAC-CGC-AAC-TC-3’ |
|  | Reverse | | 5’-TGT-TGG-CTT-TGA-CAA-TCT-TCA-C-3’ |
| m-*Cdh5* | Forward | | 5’-GCA-ATG-GCA-GGC-CCT-AAC-TTT-C-3’ |
|  | Reverse | | 5’-CAG-CAA-ACT-CTC-CTT-GGA-GCA-C-3’ |
| m-*Cldn5* | Forward | | 5’-TGA-CTG-CCT-TCC-TGG-ACC-ACA-A-3’ |
|  | Reverse | | 5’-CAT-ACA-CCT-TGC-ACT-GCA-TGT-GC-3’ |
| m-*Gja1* | Forward | | 5’-GGT-GAT-GAA-CAG-TCT-GCC-TTT-CG-3’ |
|  | Reverse | | 5’-GTG-AGC-CAA-GTA-CAG-GAG-TGT-G-3’ |
| m-*Bax* | Forward | | 5’-AGG-ATG-CGT-CCA-CCA-AGA-AGC-T-3’ |
|  | Reverse | | 5’-TCC-GTG-TCC-ACG-TCA-GCA-ATC-A-3’ |
| m-*Tnf-α* | Forward | | 5’-GGT-GCC-TAT-GTC-TCA-GCC-TCT-T-3’ |
|  | Reverse | | 5’-GCC-ATA-GAA-CTG-ATG-AGA-GGG-AG-3’ |
| m-*Il-1α* | Forward | | 5’-GGA-GAA-GAC-CAG-CCC-GTG-TTG-CT-3’ |
|  | Reverse | | 5’-CCG-TGC-CAG-GTG-CAC-CCG-ACT-T-3’ |
| m-*Icam-1* | Forward | | 5’-AAC-TGT-GGC-ACC-GTG-CAG-TC-3’ |
|  | Reverse | | 5’-AGG-GTG-AGG-TCC-TTG-CCT-ACT-TG-3’ |
| m-*Vcam-1* | Forward | | 5’-GCC-ACC-CTC-ACC-TTA-ATT-GCT-ATG-3’ |
|  | Reverse | | 5’-TGT-GCA-GCC-ACC-TGA-GAT-CC-3’ |
| m-*Isg-15* | Forward | | 5’-CAT-CCT-GGT-GAG-GAA-CGA-AAG-G-3’ |
|  | Reverse | | 5’-CTC-AGC-CAG-AAC-TGG-TCT-TCG-T-3’ |
| m-*Col1a1* | Forward | | 5’-TCA-TCG-TGG-CTT-CTC-TGG-TC-3’ |
|  | Reverse | | 5’-GAC-CGT-TGA-GTC-CGT-CTT-TG-3’ |
| m-*Col3a1* | Forward | | 5’-GAC-CAA-AAG-GTG-ATG-CTG-GAC-AG-3’ |
|  | Reverse | | 5’-CAA-GAC-CTC-GTG-CTC-CAG-TTA-G-3’ |
| m-*p16* | Forward | | 5’-TGT-TGA-GGC-TAG-AGA-GGA-TCT-TG-3’ |
|  | Reverse | | 5’-CGA-ATC-TGC-ACC-GTA-GTT-GAG-C-3’ |
| m-*p21* | Forward | | 5’-TCG-CTG-TCT-TGC-ACT-CTG-GTG-T-3’ |
|  | Reverse | | 5’-CCA-ATC-TGC-GCT-TGG-AGT-GAT-AG-3’ |
| m-*Ccl-2* | Forward | | 5’-GCT-ACA-AGA-GGA-TCA-CCA-GCA-G-3’ |
|  | Reverse | | 5’-GTC-TGG-ACC-CAT-TCC-TTC-TTG-G-3’ |
| m-*Il-6* | Forward | | 5’-TAC-CAC-TTC-ACA-AGT-CGG-AGG-C-3’ |
|  | Reverse | | 5’-CTG-CAA-GTG-CAT-CAT-CGT-TGT-TC-3’ |

**Table S2. Antibodies and reagents used in western blot and immunostaining.**

| Target antigen | Vendors or Source | Catalog number |
| --- | --- | --- |
| CLDN5 | Invitrogen | 35-2500 |
| CDH5 | Sigma (Millipore) | MABT886 |
| CX43 | Cell Signaling | 3512S |
| PARP1 | Cell Signaling | 9542S |
| Cleaved Caspase 3 | Cell Signaling | 9664S |
| BAX | Stanta Cruz | Sc-7480 |
| p-p65 | Cell Signaling | 3033S |
| p65 | Cell Signaling | 6956S |
| IκBα | Cell Signaling | 9242S |
| p-STAT1 | Cell Signaling | 9167S |
| STAT1 | Cell Signaling | 9172S |
| Asymmetric Di-Methyl Arginine | Cell Signaling | 13522S |
| F4/80 | eBioscience | 12-4801-82 |
| HSP90 | Abcam | Ab13495 |
| β-Tubulin | Invitrogen | 32-2600 |
| GAPDH | Abfrontier | LF-PA0212 |
| Goat anti-Rabbit IgG HRP | Jackson Laboratories | 111-035-003 |
| CD31 | Invitrogen | 14-0311-85 |
| αSMA | Abcam | Ab5649 |
| proSP-C | Abcam | Ab90716 |
| DAPI | Sigma Aldrich | MBD0020 |
| PRMT1 | Invitrogen | MA5-27756 |
| γH2AX | Invitrogen | MA1-2022 |
| BrdU | Abcam | Ab6326 |
| Histone H3 | Cell Siganling | 9715 |
| p16 | Proteintech | 10883-1-AP |
| p21 | Santa Cruz | Sc-471 |
| MKI67 | Abcam | ab15580 |
| Hoechst 33342 | Invitrogen | H3570 |
| Alexa Fluor® 488 goat anti-rabbit IgG (H+L) | Invitrogen | A11008 |
| Alexa Fluor® 568 goat anti-mouse IgG (H+L) | Invitrogen | A11004 |
